# Understanding the factors controlling the photo-oxidation of natural DNA by enantiomerically pure intercalating ruthenium polypyridyl complexes through TA/TRIR studies with polydeoxynucleotides and mixed sequence oligodeoxynucleotides†

**DOI:** 10.1039/d0sc02413a

**Published:** 2020-08-06

**Authors:** Páraic M. Keane, Kyra O'Sullivan, Fergus E. Poynton, Bjørn C. Poulsen, Igor V. Sazanovich, Michael Towrie, Christine J. Cardin, Xue-Zhong Sun, Michael W. George, Thorfinnur Gunnlaugsson, Susan J. Quinn, John M. Kelly

**Affiliations:** School of Chemistry, Trinity College Dublin, The University of Dublin Dublin 2 Ireland pakeane@tcd.ie jmkelly@tcd.ie; School of Chemistry, University of Reading RG6 6AD UK; Trinity Biomedical Sciences Institute, The University of Dublin Pearse St. Dublin 2 Ireland; Central Laser Facility, Research Complex at Harwell, Science and Technology Facilities Council, Rutherford Appleton Laboratories OX11 0QX UK; School of Chemistry, University of Nottingham NG7 2RD UK; Department of Chemical and Environmental Engineering, The University of Nottingham Ningbo China 199 Taikang East Road Ningbo 315100 China; School of Chemistry, University College Dublin Dublin 4 Ireland susan.quinn@ucd.ie

## Abstract

Ruthenium polypyridyl complexes which can sensitise the photo-oxidation of nucleic acids and other biological molecules show potential for photo-therapeutic applications. In this article a combination of transient visible absorption (TrA) and time-resolved infra-red (TRIR) spectroscopy are used to compare the photo-oxidation of guanine by the enantiomers of [Ru(TAP)_2_(dppz)]^2+^ in both polymeric {poly(dG-dC), poly(dA-dT) and natural DNA} and small mixed-sequence duplex-forming oligodeoxynucleotides. The products of electron transfer are readily monitored by the appearance of a characteristic TRIR band centred at *ca.* 1700 cm^−1^ for the guanine radical cation and a band centered at *ca.* 515 nm in the TrA for the reduced ruthenium complex. It is found that efficient electron transfer requires that the complex be intercalated at a G-C base-pair containing site. Significantly, changes in the nucleobase vibrations of the TRIR spectra induced by the bound excited state before electron transfer takes place are used to identify preferred intercalation sites in mixed-sequence oligodeoxynucleotides and natural DNA. Interestingly, with natural DNA, while it is found that quenching is inefficient in the picosecond range, a slower electron transfer process occurs, which is not found with the mixed-sequence duplex-forming oligodeoxynucleotides studied.

## Abstract 2

Efficient electron transfer requires the complex to be intercalated at a G-C base-pair. Identification of preferred intercalation sites is achieved by TRIR monitoring of the nucleobase vibrations before electron transfer.

## Graphical abstract

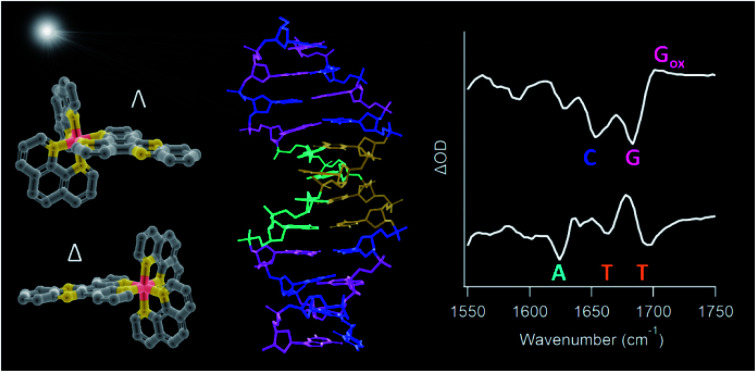

## Introduction

Ruthenium(ii) polypyridyl complexes continue to attract considerable interest due to their tuneable chemical properties and wide range of potential applications.^1^ Such complexes have shown great potential in biological systems with applications such as biomolecule recognition and cellular imaging^2^ and more recently possible phototherapeutic applications.^3^ While singlet oxygen is often posited as the reactive species generated by such ruthenium polypyridyl photosensitisers, other complexes may react through a type 1 mechanism. This is the case where the excited state of the complex is strongly oxidising, as such complexes can cause direct oxidation of proteins and nucleic acids. Examples of this are complexes containing two 1,4,5,8-tetraazaphenanthrene (TAP) ligands where it has also been demonstrated that the photoinduced electron transfer (PET) process can subsequently result in strand breaks and photo-adduct formation.^4^

**Fig. 1:**
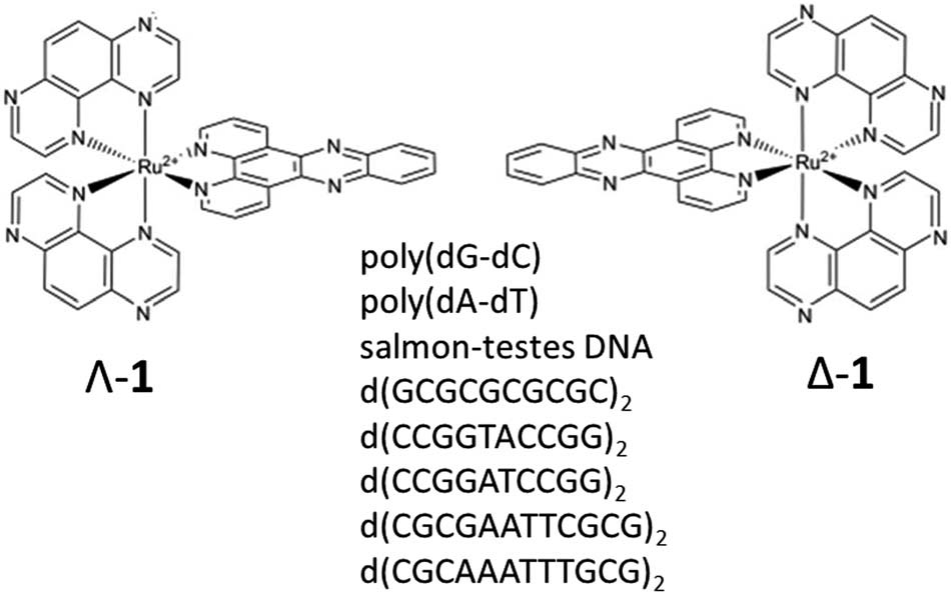
Structures of Λ-[Ru(TAP)_2_(dppz)]^2+^ (Λ-**1**), Δ-[Ru(TAP)_2_(dppz)]^2+^ (Δ-**1**) and self-complementary oligodeoxynucleotide sequences used in this study.

The photophysics and DNA-binding properties of complexes containing the dipyrido[3,2-*a*:2′,3′-*c*]phenazine (dppz) ligand have been extensively studied and [Ru(phen)_2_(dppz)]^2+^ (phen = 1,10 phenanthroline) is well known to act as a luminescence light switch where intercalation into DNA can be observed by a ‘turning-on’ of emission.^1*a*–*c*^ By contrast, [Ru(TAP)_2_(dppz)]^2+^ (**1**) (TAP = 1,4,5,8-tetraazaphenanthrene) (Fig. 1) undergoes emission quenching in the presence of DNA due to oxidation of guanine by the photo-excited complex,^5^ and related complexes are known to be highly effective at inducing photo-sensitised cell death (unlike their phen analogues).^6^ Therefore, this class of compound shows promise for the development of new DNA-targeted therapies. However, a significant challenge is to understand the mechanism of photodamage to the bio-polymer, which requires knowledge of (i) where the complex binds in a given DNA sequence, and (ii) how the photo-oxidation reactions are affected by the sequence. Additionally, each enantiomer must be examined as a distinct species, as both their binding behaviour and consequent reactivity may be expected to be different.

To address these questions we have previously reported the transient visible absorption (TrA) and time-resolved infra-red (TRIR) spectroscopy of [Ru(TAP)_2_(dppz)]^2+^ enantiomers bound to a series of short (10-mer) oligodeoxynucleotides (ODNs) comprising a range of specific AT- and GC-rich tracts.^7^ Studies with these systems have the advantage that the preference for particular sites and sequence motifs can be explored and insights into the photophysical properties in solution drawn from knowledge of the crystal structure.^8^ TRIR studies can also be carried out directly on [Ru(TAP)_2_(dppz)]^2+^–ODN crystals.^9^

The combination of these complementary spectroscopic techniques provides a powerful diagnostic approach to permit comparison of the behaviour of model synthetic systems with that of natural DNA. Transient visible absorption spectroscopy (TrA) allows one to monitor the behaviour of transient species formed from the metal complex – in particular the reaction of the excited state and the formation and subsequent decay of the reduction product [Ru(TAP)_2_(dppz)]^+^.^7^ However, the TrA technique provides little information directly about the DNA. By contrast, time-resolved infra-red spectroscopy (TRIR) allows the simultaneous monitoring of the metal complex transient species and also those formed from the DNA by interaction with the metal complex excited state.^7^ A particular strength of TRIR in such studies is that perturbation of the DNA upon formation of the metal complex excited state leads to strong absorption changes in bands characteristic of the nucleobases of the DNA, even when the DNA is not directly excited by the laser pulse (*e.g.* at 400 nm). For example, it has been shown that even when no chemical reaction takes place, excitation of an intercalated molecule can lead to transient bleaching of the DNA nucleobase vibrations. Such is the case with the light-switching complex [Ru(phen)_2_(dppz)]^2+^.^1*c*^ This “site effect” phenomenon directly provides information about the base-pair step where the metal complex is intercalated. TRIR also allows one to monitor directly the oxidation of guanine, by observing the diminution (so-called ‘bleaching’) of the absorption of the guanine vibration (centred at 1660 cm^−1^).

In comparing natural DNA with model systems it must be remembered that DNA is a polymer of mixed sequence. It is expected that short ODNs are more susceptible to the influence of end effects (but perhaps less to allosteric effects). Therefore, it is important to (i) determine if the photochemical behaviour of **1** is different in polynucleotides and ODNs and (ii) to probe its behaviour in mixed sequence systems. To this end we report here the interactions of Λ-**1** and Δ-**1** with natural DNA, synthetic polymers poly(dA-dT) and poly(dG-dC), and short ODNs containing key sequence motifs with progressive mixed sequence composition and variety of binding sites (Fig. 1). We have used steady-state spectroscopy, transient visible absorption (TrA) and time-resolved infra-red (TRIR) to (i) monitor the products and kinetics of reversible guanine photo-oxidation and (ii) to help identify the preferred binding sites in DNA. The identification of the binding site coupled with monitoring the reaction dynamics is expected to significantly contribute to our understanding of the photophysical process in polynucleotides and natural DNA.

## Results and discussion

### Binding studies with synthetic polynucleotides and natural DNA

**Fig. 2:**
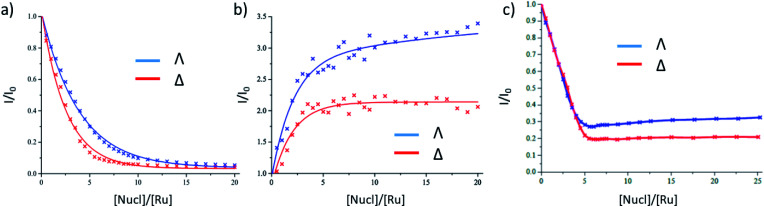
Steady-state emission titrations of Λ/Δ-[Ru(TAP)_2_(dppz)]^2+^ in the presence of (a) poly(dG-dC) (b) poly(dA-dT) (c) salmon-testes DNA. *λ*_exc_ = 435 nm; *λ*_em_ = 635 nm in 10 mM sodium phosphate buffer in H_2_O. For (a) plots are fitted to the McGhee–von Hippel equation. For (b) and (c) they are best fits using trend-line or interpolation methods. [Ru] = 20 μM (a), 8 μM (b and c).

The binding of Λ-**1** and Δ-**1** to the duplex alternating polynucleotide poly(dG-dC) was studied in air-saturated aqueous 10 mM phosphate buffer using steady-state absorption and emission spectroscopy. In the UV/Vis absorption spectra, diminution in the dppz (362 nm) and MLCT (412 nm) bands was observed, with a slight spectral shift (ESI Fig. S1a and b†). There is strong quenching of the emission at 635 nm for both enantiomers, with slightly more efficient quenching seen for Δ-**1** (Fig. 2a and ESI Fig. S1c, d†). The change of emission intensity was used to calculate association constants using the method of McGhee and von Hippel.^10^ Stronger binding was observed for Δ-**1** (*K*_b_ = 3.6 ± 0.2 × 10^6^ M^−1^, *n* = 2.2 ± 0.1 base pairs) than for Λ-**1** (*K*_b_ = 1.2 ± 0.1 × 10^6^ M^−1^, *n* = 2.0 ± 0.1 base pairs). In contrast to poly(dG-dC), binding to poly(dA-dT) results in an enhancement, rather than quenching, of emission (Fig. 2b and ESI Fig. S2†), as has previously been reported for the racemic complex.^5*a*^ While the Δ enantiomer clearly reaches saturation, this is not the case with its Λ counterpart; an indication of a particularly complicated binding for this species, as has been elegantly demonstrated by Lincoln and co-workers using isothermal calorimetry with other dppz Ru(ii)-complexes.^11^

In the presence of natural (salmon testes) DNA (st-DNA), significant quenching is observed for both enantiomers but is not complete. A larger residual emission intensity is found for the Λ enantiomer (Fig. 2c and ESI Fig. S3†). Furthermore, in the presence of the Λ enantiomer, there is a slight recovery in emission intensity at higher molar nucleotide/[Ru(TAP)_2_(dppz)]^2+^ (Nucl/Ru) ratios, again showing that a simple binding model such as that of McGhee and von Hippel is not appropriate.

### Transient spectroscopy studies in poly(dG-dC)

**Fig. 3:**
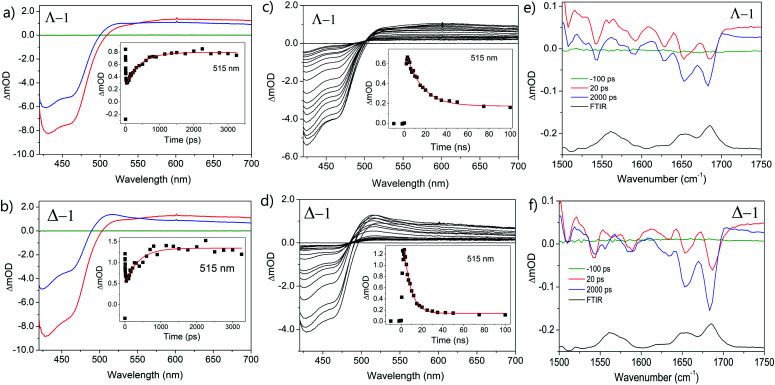
Transient spectra of Λ/Δ-[Ru(TAP)_2_(dppz)]^2+^ (400 μM) in the presence of polyd(dG-dC); (a and b) ps-TrA spectra at −100 ps (green), 20 ps (red) and 2500 ps (blue). Inset: exponential growth kinetics for reduced complex at 515 nm; (c and d) ns-TrA spectra at selected delays 0.5–100 ns (black). Inset: monoexponential fit to decay of reduced species at 515 nm; (e and f) ps-TRIR spectra at −100 ps (green), 20 ps (red), 2000 ps (blue), FTIR (black). *λ*_exc_ (psTrA/TRIR) = 400 nm, (nsTrA) = 355 nm. Pump energy = 1 μJ. In aerated 50 mM phosphate-buffered D_2_O pH 7; Nucl/Ru = 25.

**Fig. 4:**
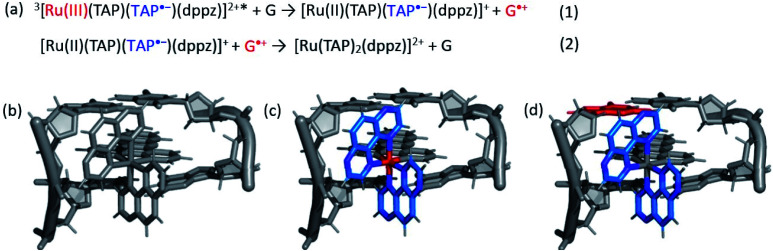
(a) Equations summarising the excited state electron transfer process of **1** in close proximity to guanine. (b) Schematic example of expected changes in electron density for Λ-**1** intercalated into a GC/GC base-pair step upon (c) formation of the excited state and (d) subsequent electron transfer. Red = oxidised centre, blue = reduced centre.

The photo-oxidation of guanine by Λ-**1** and Δ-**1** bound to poly(dG-dC) was studied by both TrA and TRIR in aerated 50 mM potassium phosphate D_2_O buffer at a Nucl/Ru ratio of 25, similar conditions to those used previously for analogous studies with ODNs and where >90% of the complex is bound to the DNA.^7^ Notably, every binding site will place the complex adjacent to a guanine. In the TrA spectra a large negative ‘bleach’ signal is observed below 500 nm immediately after laser excitation (400 nm), corresponding to removal of the ground state absorption (Fig. 3a and b). A broad transient feature above 500 nm (*λ*_max_*ca.* 600 nm), which may be assigned as the ^3^MLCT* excited state [Ru(iii)(TAP)(TAP˙^−^)(dppz)]^2+^*, is also present. As time evolves, a band grows in at 515 nm, assigned by spectro-electrochemical experiments and DFT calculations^12^ to the reduced complex [Ru(ii)(TAP)(TAP˙^−^)(dppz)]^+^ formed by photoinduced transfer of an electron from a close lying guanine (Fig. 4, eqn (1)).

The yield of ET, as seen by the strength of the band at 515 nm, is larger for Δ-**1** than for Λ-**1**. The rate of forward ET was determined by fitting the growth of this band to exponential kinetics, giving values of *ca.* 500 ps in both cases (Table 1), similar to the value previously reported with the racemic compound.^5*b*^ The rate of back ET (eqn (2)) was determined by monitoring the subsequent decay of the reduced ruthenium complex by ns-TrA (355 nm excitation) (Fig. 3c and d), giving lifetime values of 14 ± 2 ns and 8 ± 1 ns for Λ-**1** and Δ-**1**, respectively.^13^

**Table 1:** Photophysical parameters for Λ-**1** and Δ-**1** in the presence of poly(dG-dC) in 50 mM phosphate buffered D_2_O

	Λ-**1**	Δ-**1**
Forward ET, TrA (ps)^a^	570 ± 60	520 ± 50
Forward ET, TRIR (ps)^b^	680 ± 180	490 ± 80
Back ET, TrA (ns)^a^	14.1 ± 2.0	7.7 ± 0.8
Back ET, TRIR (ns)^b^	14.9 ± 2.0	6.5 ± 1.0

a Measured at 510 nm.

b Measured at 1680 cm^−1^.

Unlike visible TrA spectroscopy, TRIR may report on the changes in the DNA as well as those of the complex.^7,14^ The TRIR spectra for **1** contains both transient absorption and ‘bleach’ vibrational bands at wavenumbers below 1550 cm^−1^ (Fig. S5†) but only very weak features between 1550 cm^−1^ and 1750 cm^−1^. However, when the metal complex is excited (at 400 nm) in the presence of poly(dG-dC) strong ‘bleaches’ are observed, which may be assigned to the loss of the ground state vibrations of the constituent nucleotides (Fig. 3e, f and S5†). The most prominent of these bands are at 1650 cm^−1^ (C carbonyl) and 1680 cm^−1^ (G carbonyl). Note that DNA does not absorb at 400 nm (Fig. S6†) so that the appearance of these bands is not caused by the direct excitation of poly(dG-dC). These bands, which emerge before the electron transfer takes place, are attributed to the “site effect” and are proposed to be produced due to an electronic perturbation of the neighbouring nucleobases by the photo-excited complex, see Fig. 4c, and to therefore provide information about the binding site. Interestingly the bleaching profile is different for each enantiomer, with a larger relative intensity of the G band (1680 cm^−1^) for the Δ enantiomer, presumably indicating differences of the binding site.

At longer times (up to *ca.* 1 ns) the bleaching of the G and C bands increase further in magnitude, which can be attributed to chemical reaction of the guanine. The growth of these bleaching bands was fitted to an exponential function, giving similar rates of growth to those observed by ps-TrA for the reduction of the metal complex (Table 1), as expected for the PET process. A small transient feature also appears at *ca.* 1700 cm^−1^ for either enantiomer bound to poly(dG-dC). This is a characteristic marker band of oxidised guanine.^15^

The transient spectra and kinetic parameters found for **1** bound to poly(dG-dC) are similar to those observed for **1** bound to the duplex ODN d(GCGCGCGCGC)_2_.^7*a*^ (ESI Fig. S7 and Table S1†). This shows that in this case the photophysical/photo-chemical behaviour are closely comparable for oligomers or polymers and that ODNs may be considered a good model for their macromolecular equivalents.

### Transient spectroscopy studies in poly(dA-dT)

The binding of either enantiomer to poly(dA-dT) leads to strong luminescence enhancement, as demonstrated in Fig. 2 and ESI Fig. S2,† which is consistent with the excited state not being quenched. (This enhancement is probably primarily due to protection of the bound-complex from oxygen in the aerated solution).^5*a*^

**Fig. 5:**
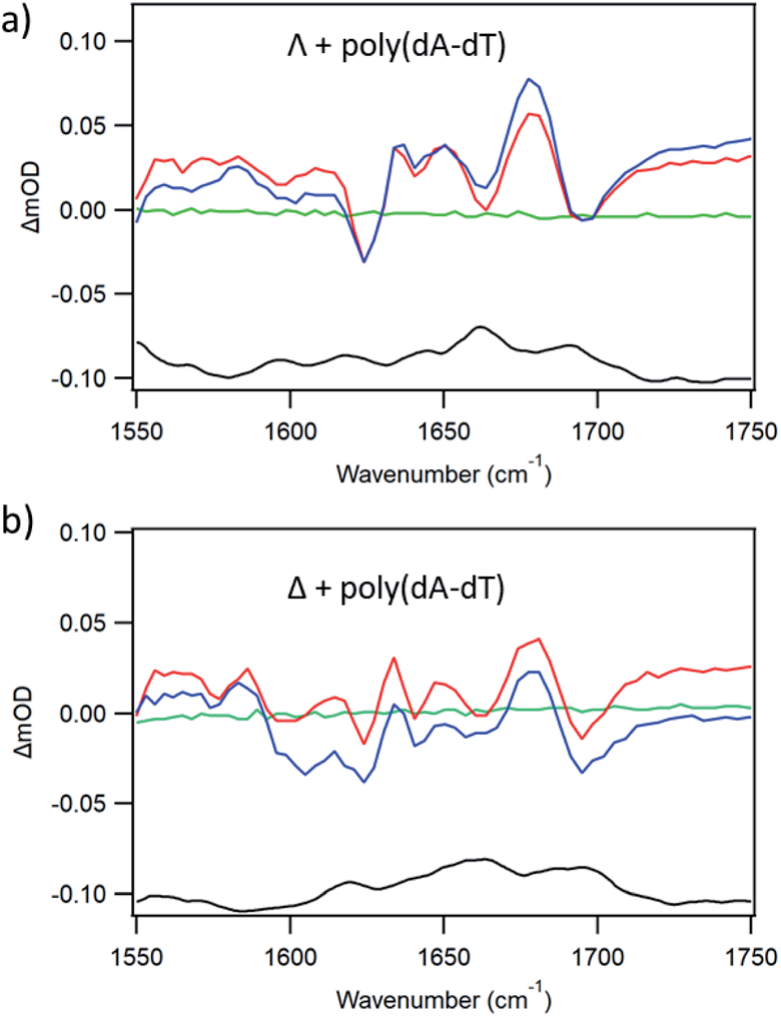
TRIR spectra of 400 μM (a) Λ- and (b) Δ-[Ru(TAP)_2_(dppz)]^2+^ with poly(dA-dT), Nucl/Ru = 15, in aerated 50 mM phosphate-buffered D_2_O pH 7. Spectra shown at −100 ps (green) 20 ps (red) 2000 ps (blue) FTIR (black) (*λ*_exc_ = 400 nm, 1 μJ).

The TRIR spectra recorded for Λ-**1** and Δ-**1** bound to poly(dA-dT) are shown in Fig. 5. The excitation of the bound complex (at 400 nm) gives rise to distinctive features in the DNA region (>1550 cm^−1^). Prominent bleach bands are observed for both enantiomers at 1700 cm^−1^, 1660 cm^−1^ and 1625 cm^−1^ and are consistent with vibrations of the thymine carbonyl and adenine groups.^7*b*,16^ Some variation is observed in the intensity of the bleach bands observed for Λ-**1** and Δ-**1**, which may arise due to the different intercalation orientations that are predicted for these enantiomers when bound to poly(dA-dT).^17^ The considerable difference in the TRIR spectra from those shown in Fig. 3 in the presence of poly(dG-dC) is an example of the advantage of the “site effect” in reporting on the DNA environment in the proximity of the excited state of **1**.

### Transient spectroscopy studies in natural (salmon testes) DNA

**Fig. 6:**
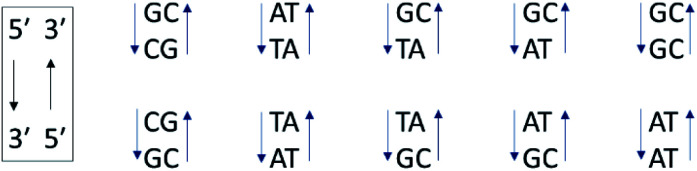
The ten Watson–Crick base-pair steps in natural DNA. Their sequence notation is written in the box.

**Fig. 7:**
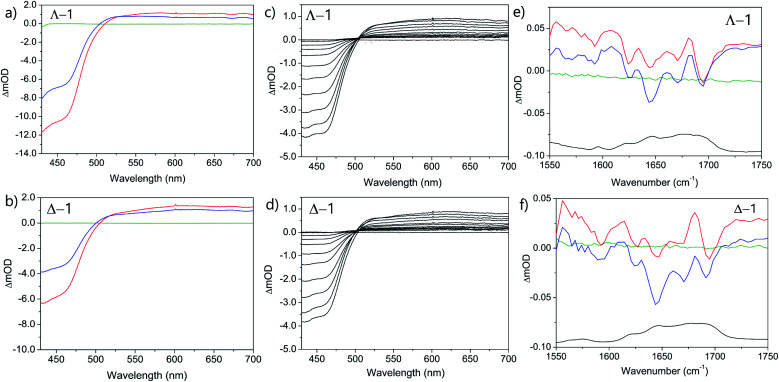
Transient spectra of Λ/Δ-[Ru(TAP)_2_(dppz)]^2+^ in the presence of st-DNA (a and b) ps-TrA spectra at −100 ps (green), 20 ps (red), 2500 ps (blue); (c and d) ns-TrA spectra at selected delays 2–5000 ns; (e and f) ps-TRIR spectra at −100 ps (green), 20 ps (red), 2500 ps (blue), FTIR (black); *λ*_exc_ (ps-TrA/TRIR) = 400 nm, (ns-TrA) = 355 nm. Pump energy = 1 μJ. In 50 mM phosphate-buffered D_2_O pH 7; Nucl/Ru = 25.

In contrast to the above homopolymers which each have only 2 types of base-pair steps, st-DNA, which contains roughly 42% GC and 58% AT base-pairs, has ten (Fig. 6) and therefore a much larger number of potential intercalation sites. If one of the base-pairs at the intercalation site contains guanine, efficient photo-induced electron transfer (PET) may be expected. We have therefore performed TrA/TRIR experiments for Λ-**1** and Δ-**1** bound to st-DNA (Fig. 7) in order to (i) assess the extent of guanine photo-oxidation in such natural DNA sequences and (ii) see whether TRIR can offer insight into any preferred binding sites of the complex, based on the differing bleached IR profiles of GC and AT base-pairs. Additionally, in order to gain further insights into these experiments with natural DNA we report the TRIR/TrA of some mixed sequence ODNs in the next section.

**Fig. 8:**
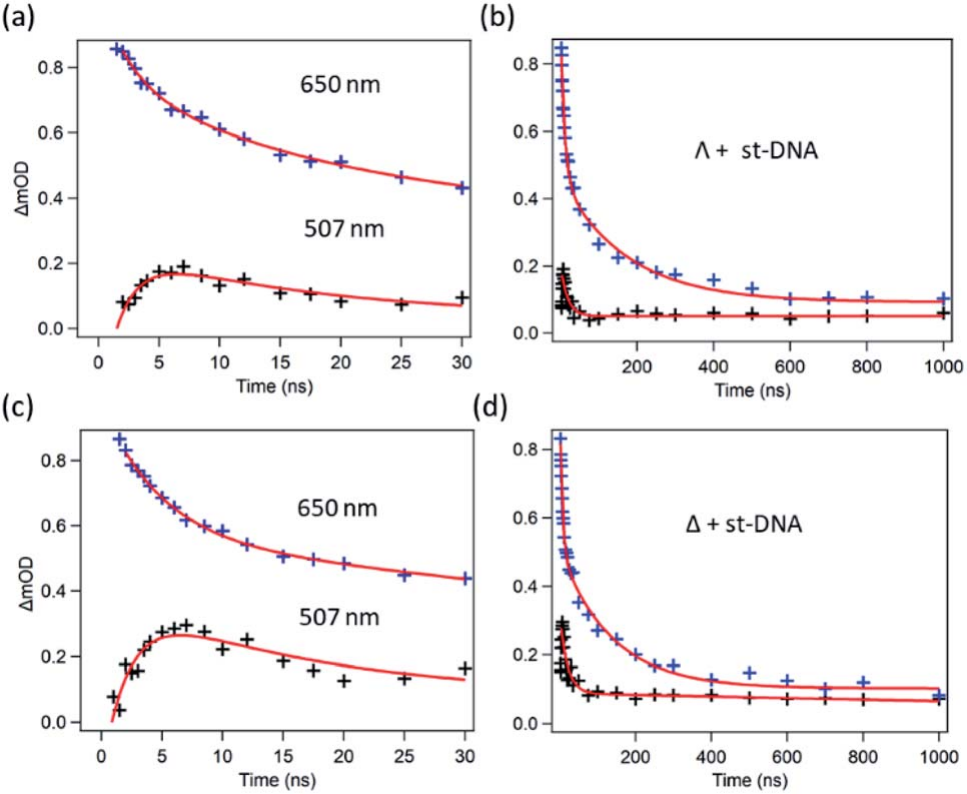
Ns-TrA kinetics for (a and b) Λ-[Ru(TAP)_2_(dppz)]^2+^ st-DNA (c and d) Δ-[Ru(TAP)_2_(dppz)]^2+^ st-DNA at short (2–30 ns) and long (to 1000 ns) time delays, recorded at 507 nm (ref. 18) (black) and 650 nm (blue). Red fitted lines are exponential functions.

TrA experiments carried out with either enantiomer in the presence of st-DNA (Fig. 7a and b) show only a modest growth over the picosecond time scale of the transient absorption at 515 nm, which is associated with the reduced metal complex. However, unlike what was observed with poly(dG-dC), nanosecond TrA (Fig. 8) reveals a slower formation of some reduced species (lifetime 2.0 ± 0.5 ns) for both Λ-**1** and Δ-**1**.^18^ This species subsequently decays with a major lifetime component of 14 ± 2 and 13 ± 2 ns for Λ-**1** and Δ-**1** respectively. The decay at 650 nm (where the excited state absorbs) appears to contain a number of components (Fig. 8b and d); biexponential fitting from 25 ns onwards gives a substantial amount of long-lived absorption, which decays with a lifetime of 600 ± 90 ns (Λ-**1**) and 680 ± 170 ns (Δ-**1**) (ESI Fig. S8 and Table S2†).

TRIR spectra in the presence of st-DNA recorded at 20 and 2000 ps reveal that both enantiomers behave similarly (Fig. 7e and f). Bleach signals at *ca.* 1680 cm^−1^ (expected for guanine) are relatively weak, while a prominent bleach signal is observed at 1695 cm^−1^, similar to that found for **1** bound to poly(dA-dT) and assigned to the thymine C2=O2 vibration. (It may be observed that the TRIR is much better resolved than the FTIR, as the TRIR is expected to only report on the binding site of the complex while the FTIR contains signals from all nucleobases in the DNA).

It is instructive to compare the experimentally observed TRIR spectra with those obtained from a combination of **1** bound to either poly(dA-dT) and poly(dG-dC) in the ratio of 58 : 42 (as found for the nucleotides in the natural polymer) (Fig. S9†). The spectra show similar bands, but there are significant differences in band intensities, which probably indicates that binding sites other than the four found in the homopolymers are occupied.

### Binding and transient studies with oligodeoxynucleotides (ODNs) of mixed sequence

To try to identify such binding sites (and their role in photo-oxidation) we next carried out TrA/TRIR experiments with Λ-**1** and Δ-**1** in the presence of various double-stranded ODNs, which contain many of the base-pair steps expected in natural DNA (as shown in Fig. 6). In general, these ODNs have a terminal GC-rich segment and an AT-rich core.

Initially we compared the behaviour of the enantiomers of **1** bound to either d(CCGGTACCGG)_2_ or d(CCGGATCCGG)_2_, which differ only in the order of the bases in the central base-pair step. For Δ-**1** the steady state titrations, transient spectra and kinetics are similar for either ODN (although the yield of electron transfer is slightly higher in the sequence with the central AT sequence) (ESI Fig. S10 and S11†). For both ODNs TRIR spectra show strong bleaching at *ca.* 1650 and 1680 cm^−1^ as expected for photo-oxidation of the ODN (ESI Fig. S11†). This behaviour contrasts markedly with that of Λ-**1**, with which we previously reported stronger binding, lower yields of ET and a much slower ns decay in the presence of d(CCGGTACCGG)_2_ than in the ODN with the central AT/AT base-pair step (ESI Fig. S12†).^7*b*^ This demonstrates that the Λ-enantiomer has a strong preference for TA/TA base-pair steps, a selectivity which is not expressed by its Δ-counterpart.

We next examined d(CGCGAATTCGCG)_2_ and d(CGCAAATTTGCG)_2_ as targets. These ODNs, which have a more extended AT-rich central segment, were selected as they have also been widely used as models for DNA structure and in binding studies.^19^

**Fig. 9:**
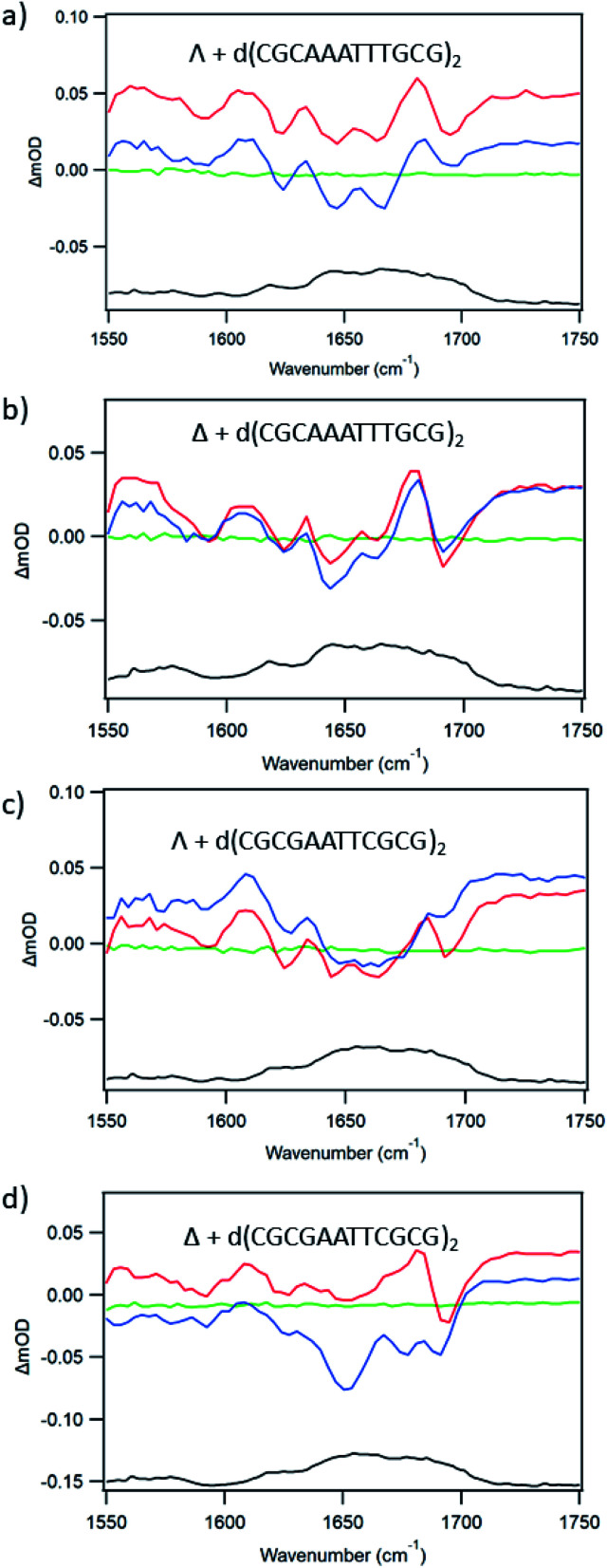
Ps-TRIR spectra of Λ/Δ-[Ru(TAP)_2_(dppz)]^2+^ in the presence of dodecamer ODNs: (a) Λ + d(CGCAAATTTGCG)_2_ (b) Δ + d(CGCAAATTTGCG)_2_ (c) Λ + d(CGCGAATTCGCG)_2_ (d) Δ + d(CGCGAATTCGCG)_2_. Recorded at −100 ps (green), 20 ps (red) 2500 ps (blue), FTIR (black). In 50 mM phosphate-buffered D_2_O pH 7. *λ*_exc_ = 400 nm, 1 μJ.

For d(CGCAAATTTGCG)_2_ the plot of emission intensity upon addition of the ODN (ESI Fig. S13a, c and e†) is complex for both enantiomers with a significant reduction of the intensity initially and then an increase subsequently. Fig. 9a and b shows the ps-TRIR spectra recorded at 20 ps and 2000 ps for the enantiomers of **1** bound to this ODN (at Nucl/Ru = 25). It may be observed for both enantiomers that the spectra do not change greatly over this time period, suggesting that any ET reaction occurring on this timescale must do so with a small yield. In agreement with this, the average lifetime recorded for the transients observed by ns-TrA (ESI Fig. S14 and Tables S3, S4†) is >100 ns. These measurements are consistent with each of the enantiomers binding in the 5′-AAATTT segment, probably at the AA/TT base-pair step, so that the complex is not in contact with a guanine.

For d(CGCGAATTCGCG)_2_, the ODN with the shorter AT run and hence more GC, both enantiomers of **1** show substantial and increasing quenching of the steady state luminescence upon addition of the ODN (ESI Fig. S13b, d and f†). In this case (at Nucl/Ru = 25) the changes in the ps-TRIR between 20 and 2000 ps are rather small for Λ-**1** (Fig. 9c) For Δ-**1** the change is more significant (Fig. 9d), which would occur if some of the Δ-enantiomers bound close to a guanine moiety, such as the GA/TC base-pair step.

**Fig. 10:**
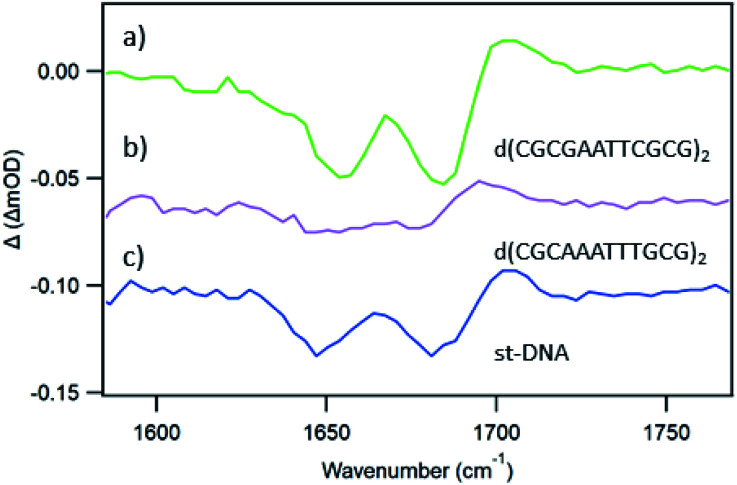
TRIR subtraction spectra (2000 ps minus 20 ps) for Δ-[Ru(TAP)_2_(dppz)]^2+^ in the presence of mixed sequence DNAs (a) d(CGCGAATTCGCG)_2_, (b) d(CGCAAATTTGCG)_2_, (c) st-DNA.

In an effort to establish the contribution from electron transfer when **1** is intercalated into these ODNs, the signal obtained at 20 ps was subtracted from that at 2000 ps. This procedure was adopted because, as noted previously, the signal for a photo-oxidised base-pair site grows in over this time period, while the signal due solely to the effect of the excited state on the nucleobases at the binding site is present very shortly after excitation. Using this treatment, it may be observed (Fig. 10a) that the resulting subtraction spectrum for Δ-**1** + d(CGCGAATTCGCG)_2_ resembles the spectrum in poly(dG-dC). The greater strength of the bleaches in Δ-**1** in the presence of d(CGCGAATTCGCG)_2_ contrasts strongly with the absence of this feature for Δ-**1** with d(CGCAAATTTGCG)_2_ (Fig. 10b) and is consistent with the higher yield of ET in the former case. Similar treatment of the data from st-DNA (Fig. 10c) also indicates the presence of some photo-oxidation. Comparable subtraction spectra for the Λ complex are presented in ESI Fig. S15.†

## Conclusions

The experiments reported above, use a combination of both TrA and TRIR to demonstrate how the efficiency of electron transfer depends on both the enantiomer used and on the sequence of the binding site. Importantly we show how data obtained from both polymeric and oligomeric synthetic DNAs can be utilised to help determine how **1** interacts with natural DNA.

TrA is particularly useful at following the ET process (by monitoring the formation of the reduced ruthenium complex), while TRIR allows one not only to monitor the oxidation of the guanine but also provides information on the nature of the binding site. This is achieved through recording the TRIR spectrum (in the DNA nucleobase region) before electron transfer has occurred. The cause of this phenomenon is not yet completely elucidated. One possibility that can be considered is that it is caused by a temperature jump effect. Transient changes in the IR spectra of ODNs caused by a temperature jump initiated by high energy laser pulses have been reported recently by Hunt and co-workers.^20^ These researchers observed that the temperature jump could cause melting or pre-melting of the ODN. However, the kinetics reported are quite different from what is observed in our studies. Provisionally we attribute the “site effect” observed to a type of vibrational Stark effect,^21^ induced by the changes of electron density in [Ru(TAP)_2_(dppz)]^2+^ upon formation of its excited state.

Electron transfer is noted with poly(dG-dC), but not with poly(dA-dT), consistent with guanine being the target nucleobase. In poly(dG-dC), the reaction occurs with both enantiomers although the yield of transient photoproduct is greater for Δ compared to Λ. The differing yields of the two enantiomers is expected to be a consequence of the differing orientation of the complex in the binding site. Closely similar observations are found with d(GCGCGCGCGC)_2_, indicating that (at least in this case) the electron transfer reaction proceeds similarly in poly- or oligo-deoxynucleotides. This implies that end effects, as could be present in the short sequence, or allosteric binding, which are more likely in the polymers, have little effect on the observed photo-oxidation processes for these alternating GC systems.

For either enantiomer, although stronger binding is observed to natural DNA than to poly(dG-dC), photo-induced electron transfer is less efficient. This is consistent with preferred binding at AT-rich sites, a finding clearly demonstrated by the TRIR spectrum. From both spectroscopic and X-ray crystallography it is expected that binding for the lambda complex will occur in 5′-pyrimidine-purine-3′ base-pair steps with preference in the order TA/TA > TG/CA > CG/CG sites.^7*d*,8*b*^ TRIR spectra of Λ-**1** in the presence of d(CGCAAATTTGCG)_2_ also reveal that the complex can bind strongly at an AA/TT site and shows that this is competitive with binding to a TG/CA step. For the delta enantiomer the situation is less clear, as there are fewer crystallographic studies,^22^ but our transient studies with d(CCGGTACCGG)_2_ clearly reveal that Δ-**1** does not show the same strong preference for TA/TA steps.

**Fig. 11:**
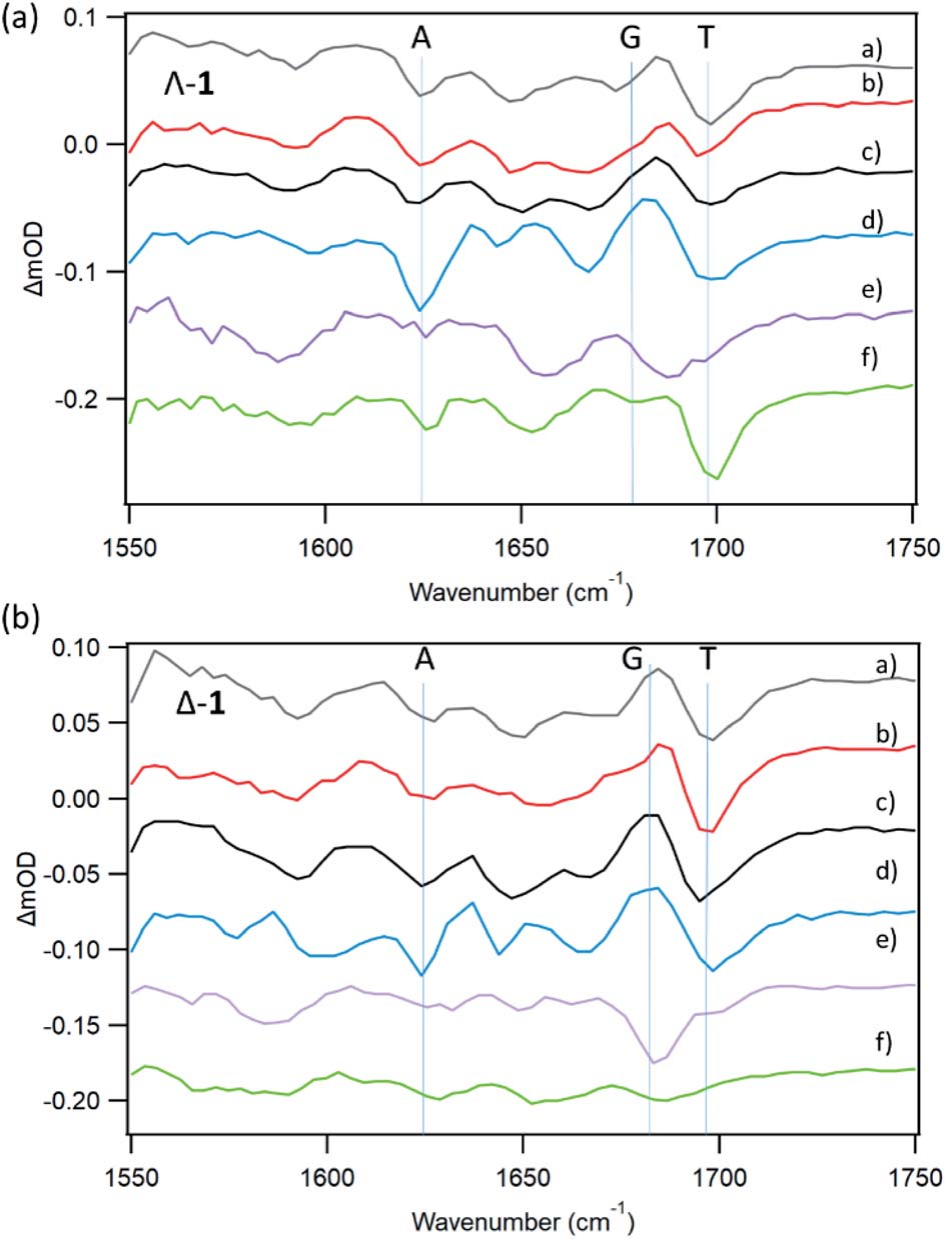
TRIR spectra (20 ps) for Λ-**1** and Δ-**1** in the presence of AT-containing mixed/defined sequence DNAs (a) st-DNA (b) d(CGCGAATTCGCG)_2_ (c) d(CGCAAATTTGCG)_2_ (d) poly(dA-dT) (e) d(CCGGATCCGG)_2_ (f) d(CCGGTACCGG)_2_. Selected bleach vibrations highlighted.

With natural DNA a striking result is that although the steady state luminescence of both enantiomers of **1** are strongly (though incompletely) quenched on binding, the TrA measurements at 2500 ps reveal low initial PET yields. TRIR spectra at 20 ps show bleach patterns for both enantiomers consistent with perturbation at AT-rich sites. Comparison with other AT containing systems are shown in Fig. 11. It may be noted that the best match for Δ-**1** is with d(CGCAAATTTGCG)_2_ which suggests that a major binding site in the natural DNA for this enantiomer could be an AA/TT step, while for Λ-**1** features suggesting binding to AA/TT or TA/TA are apparent.

Intriguingly, and in contrast to what was found with the various ODNs studied (ESI Fig. S16†), measurements for the formation of [Ru(TAP)_2_(dppz)]^+^ reveal that there is a slower PET process. While the origin of this process is not definitively known, it is possible that this could be caused by semi-intercalation of the TAP groups into a GG dinucleotide segment of the DNA remote from the intercalated complex, similar to what is found in crystals.^23^ Such a process, which would require looping of the DNA, was recently reported by Vanderlinden *et al.* for other Ru–TAP compounds.^24^

Finally, the differing reactivity of the Λ and Δ enantiomers and their ability to preferentially target AT regions (such as are found in the TATA box^25^) should be noted, given the growing interest in using ruthenium polypyridyls as photo-therapeutic agents.

## Experimental

TrA/TRIR data were recorded at the ULTRA apparatus at the Lasers for Science Facility, Rutherford Appleton Laboratories (UK).^26^ Further details, complex synthesis and data analysis methods are to be found in the ESI.†

## Conflicts of interest

There are no conflicts to declare.

## Supplementary Material

SC-011-D0SC02413A-s001
