# Supplementary material for: Understanding the factors controlling the photo-oxidation of natural DNA by enantiomerically pure intercalating ruthenium polypyridyl complexes through TA/TRIR studies with polydeoxynucleotides and mixed sequence oligodeoxynucleotides
Source: Chem Sci. 2020 Aug 6;11(32):8600–9. doi: 10.1039/d0sc02413a (PMC8163394; doi:10.1039/d0sc02413a)
Supplement: SC-011-D0SC02413A-s001 [file SC-011-D0SC02413A-s001.pdf]

Electronic Supplementary Material (ESI) for Chemical Science.
This journal is © The Royal Society of Chemistry 2020

Electronic Supporting Information

Understanding the factors controlling the photo-oxidation of natural DNA by enantiomerically pure intercalating ruthenium polypyridyl complexes through TA/TRIR studies with polydeoxynucleotides and mixed sequence oligodeoxynucleotides

Páraic M. Keane,[ab]* Kyra O'Sullivan,[a] Fergus E. Poynton, [ac] Bjørn C. Poulsen,[ac] Igor V. Sazanovich,[d] Michael Towrie,[d] Christine J. Cardin,[b] Xue-Zhong Sun,[e] Michael W. George,[ef] Thorfinnur Gunnlaugsson,[ac] Susan J. Quinn[g]* and John M. Kelly[a]*

[a] *School of Chemistry, Trinity College Dublin, The University of Dublin, Dublin 2, Ireland*
email: keanepa@tcd.ie; jmkelly@tcd.ie
[b] *School of Chemistry, University of Reading, RG6 6AD, UK*
[c] *Trinity Biomedical Sciences Institute, The University of Dublin, Pearse St., Dublin 2, Ireland*
[d] *Central Laser Facility, Research Complex at Harwell, Science and Technology Facilities Council, Rutherford Appleton Laboratories, OX11 0QX, UK*
[e] *School of Chemistry, University of Nottingham, NG7 2RD, UK*
[f] *Department of Chemical and Environmental Engineering, The University of Nottingham Ningbo China, 199 Taikang East Road, Ningbo 315100, China*
[g] *School of Chemistry, University College Dublin, Dublin 4, Ireland*
email: susan.quinn@ucd.ie

Figures

Fig. S1. UV/vis and luminescence spectra of Λ-[Ru(TAP)$_2$(dppz)]$^{2+}$ (Λ-**1**) and Δ -[Ru(TAP)$_2$(dppz)]$^{2+}$ (Δ-**1**) in the presence of increasing concentrations of poly(dG-dC) in 10 mM phosphate buffer.

Fig. S2. UV/vis and luminescence spectra of Λ-**1** and Δ-**1** in the presence of increasing concentrations of poly(dA-dT) in 10 mM phosphate buffer.

Fig. S3. UV/vis and luminescence spectra of Λ-**1** and Δ-**1** in the presence of increasing concentrations of salmon-testes DNA in 10 mM phosphate buffer.

Fig. S4. Ns-TrA kinetic plots for of Λ-**1** in the presence of poly(dG-dC) in 10 mM phosphate buffer.

Fig. S5. Comparison of TRIR spectra of Λ-**1** (alone) and Λ-**1** + poly(dG-dC).

Fig. S6. UV/vis spectra of Λ-**1**, d(CGCAAATTTGCG)$_2$ and Λ-**1** + d(CGCAAATTTGCG)$_2$.

Fig. S7. Ps-TRIR spectra of Λ-**1** and Δ-**1** in the presence of d(GCGCGCGCGC)$_2$.

Fig. S8. Biexponential fits at 650 nm for (a) Λ-**1** + st-DNA (b) Δ-**1** + st-DNA. Delays fitted after 25 ps.

Fig. S9. Ps-TRIR spectra (a) Λ-**1** + st-DNA (b) linear combination 58% {Λ-**1** + poly(dA-dT)} + 42% {Λ-**1** + poly(dG-dC)} (c) Δ-**1** + st-DNA (d) 58% {Δ-**1** + poly(dA-dT)} + 42% {Δ-**1** + poly(dG-dC)}.

Fig. S10. Changes in the molar absorption coefficient and emission intensity of Δ-**1** with increasing additions of d(CCGGATCCGG)$_2$ and d(CCGGTACCGG)$_2$ in 50 mM phosphate buffer.

Fig. S11. Ns-TrA and ps-TRIR spectra of Δ-**1** in the presence of d(CCGGTACCGG)$_2$ and d(CCGGATCCGG)$_2$ in 50 mM phosphate buffer in D_2O.

Fig. S12. Ps- & ns-TrA and ps-TRIR of Λ-**1** in the presence of d(CCGGATCCGG)$_2$ or d(CCGGTACCGG)$_2$ in 50 mM phosphate-buffered D_2O [modified from Keane *et al. Angew. Chem. Int Ed.*, 2015, **54**, 8364–8368].

Fig. S13. Changes in luminescence intensity of Λ/Δ-**1** in the presence of d(CGCAAATTTGCG)$_2$ and d(CGCGAATTCGCG)$_2$ with corresponding UV/vis and luminescence spectra in 50 mM phosphate buffer.

Fig. S14. Ps-TrA and ns-TrA spectra of Λ/Δ-**1** in the presence of d(CGCGAATTCGCG)$_2$ and d(CGCAAATTTGCG)$_2$ in 50 mM phosphate buffer in D_2O.

Fig. S15. TRIR subtraction spectra (2000 ps minus 20 ps) for Λ-**1** in the presence of d(CGCGAATTCGCG)$_2$, d(CGCAAATTTGCG)$_2$ and st-DNA in 50 mM phosphate buffer in D_2O.

Fig. S16. Comparison of Ns-TrA kinetics at 507 nm of Λ/Δ-**1** to selected ODN and polynucleotide systems.

Tables

Table S1. Comparison of kinetics obtained from ps/ns-TrA data for Λ/Δ-**1** in the presence of poly(dG-dC)$_2$ and d(GCGCGCGCGC)$_2$.

Table S2. Exponential fits to ns-TrA spectra of Λ/Δ-**1** in the presence of st-DNA.

Table S3. Average lifetimes (515 nm) for Λ/Δ-**1** in the presence of mixed sequence DNA(s).

Table S4. Average lifetimes (650 nm) for Λ/Δ-**1** in the presence of mixed sequence DNA(s).

Experimental

References

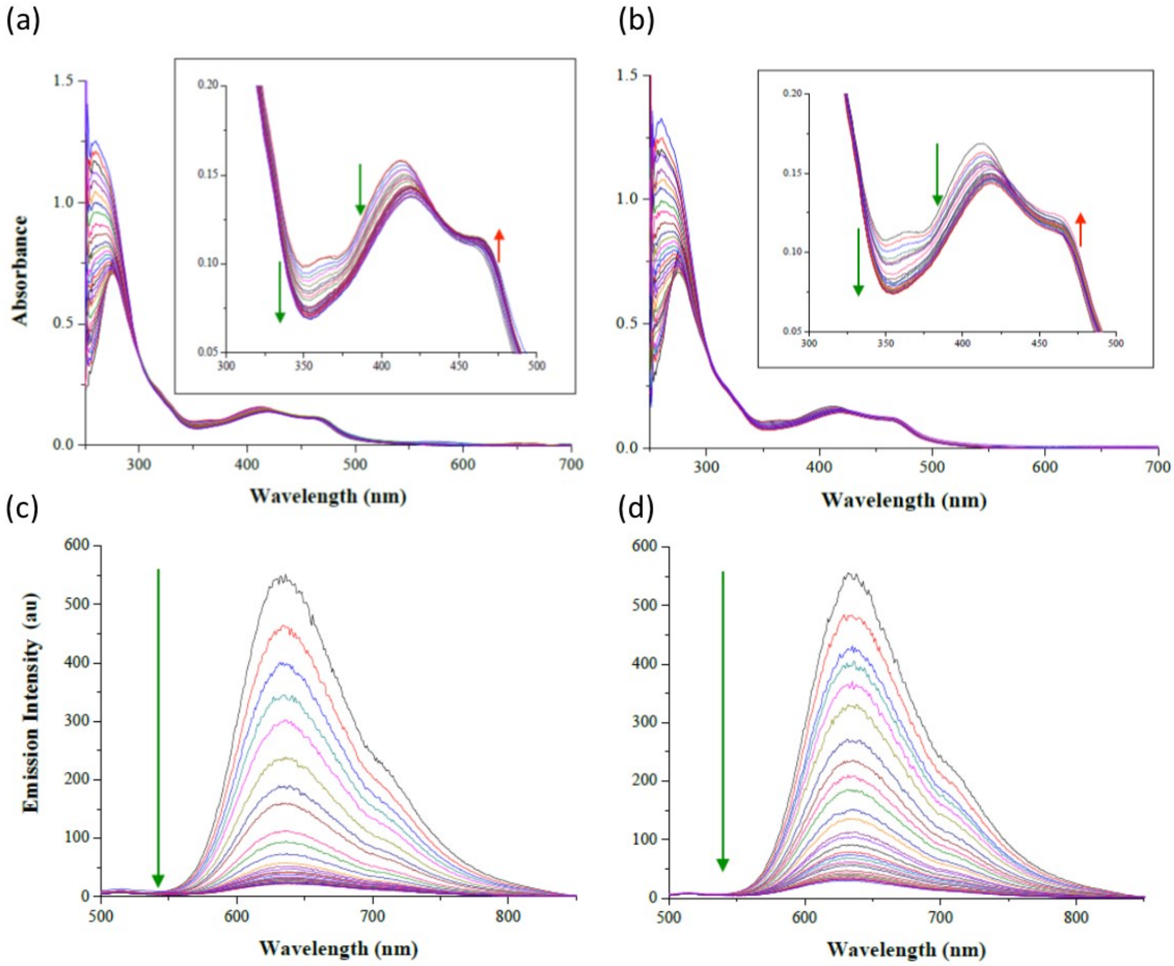

Fig. S1. UV/vis spectra of (a) Λ-$[Ru(TAP)_2(dppz)]^{2+}$ (Λ-**1**) (b) Δ-$[Ru(TAP)_2(dppz)]^{2+}$ (Δ-**1**) in the presence of increasing concentrations of poly(dG-dC) in aerated 10 mM potassium phosphate buffer (pH 7). Inset: expanded MLCT region. Luminescence spectra of (c) Λ-**1** (d) Δ-**1** in the presence of increasing concentrations of poly(dG-dC). λ_{exc} = 435 nm. [Ru] = 8 μM.

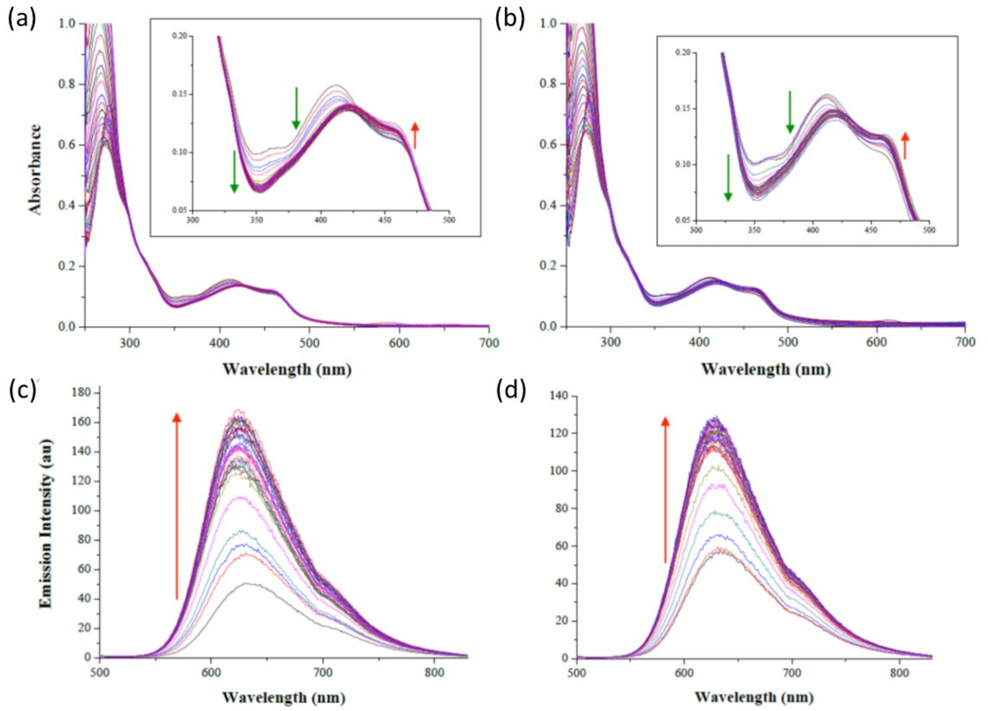

Fig. S2. UV/vis spectra of (a) Λ-**1** (b) Δ-**1** in the presence of increasing concentrations of poly(dA-dT) in aerated 10 mM potassium phosphate buffer (pH 7). Inset: expanded MLCT region. Luminescence spectra of (c) Λ-**1** (d) Δ-**1** in the presence of increasing concentrations of poly(dA-dT). λ_{exc} = 435 nm. [Ru] = 8 μM.

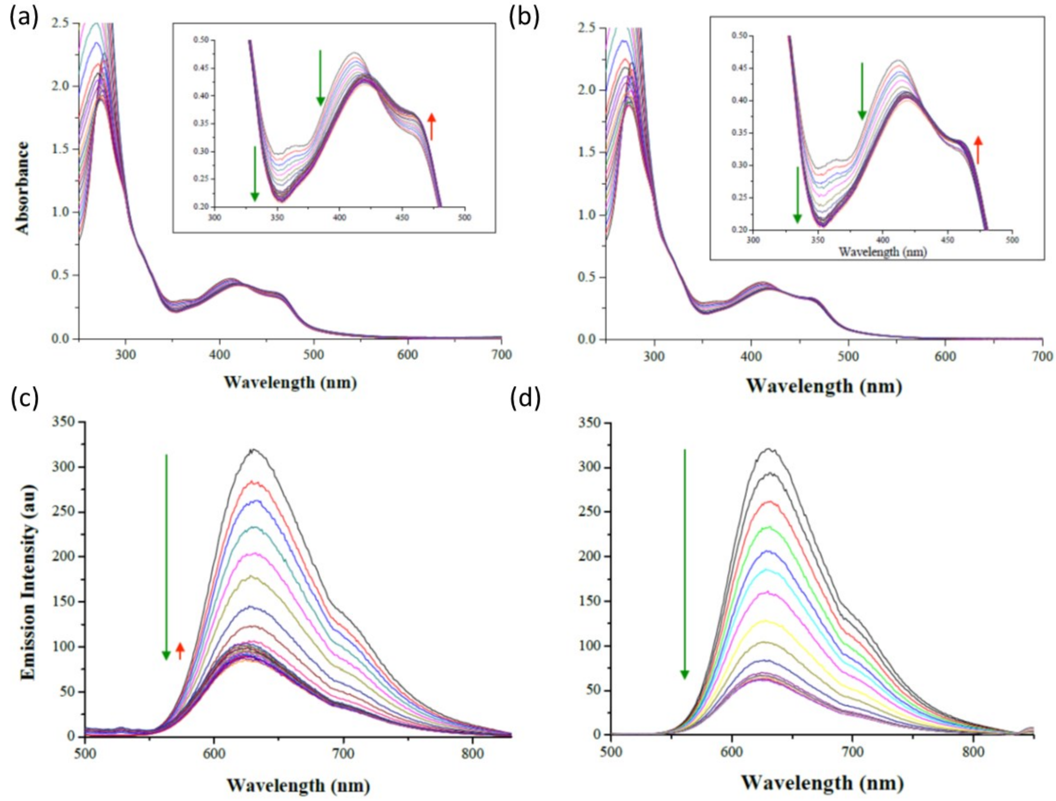

Fig. S3. UV/vis spectra of (a) Λ-**1** (b) Δ-**1** in the presence of increasing concentrations of st-DNA in aerated 10 mM potassium phosphate buffer (pH 7). Inset: expanded MLCT region. Luminescence spectra of (c) Λ-**1** (d) Δ-**1** in the presence of increasing concentrations of st-DNA. λ_{exc} = 435 nm.

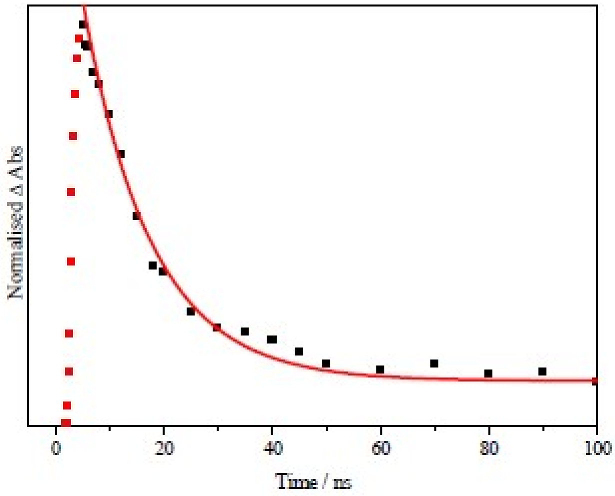

Fig. S4. Ns-TrA kinetics (510 nm) for Λ-**1** in the presence of poly(dG-dC) in 10 mM phosphate buffer pH 7 in D_2O. τ = 14 ± 0.5 ns.

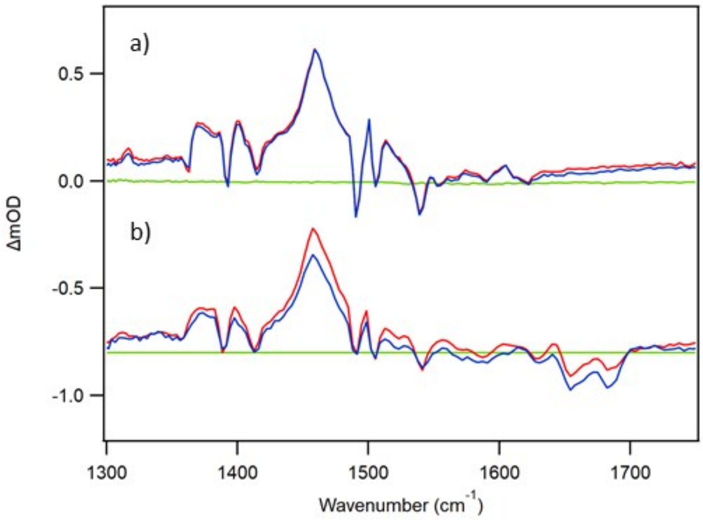

Fig. S5. Ps-TRIR spectra of (a) Λ-**1** (b) Λ-**1** + poly(dG-dC) (P/D = 20, [Ru] = 400 μM) in 50 mM phosphate-buffered D_2O pH 7. red = 20 ps, blue = 2000 ps.

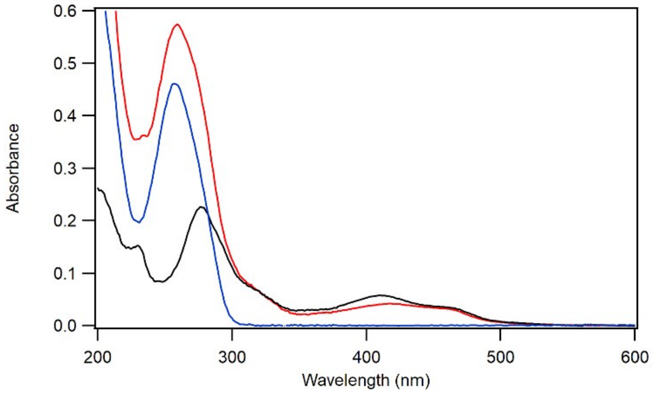

Fig. S6. UV/vis spectra of Λ-**1** (black), d(CGCAAATTTGCG)$_2$ (blue), Λ-**1** + d(CGCAAATTTGCG)$_2$ (red) in 50 mM phosphate-buffered D_2O. [Ru] = 400 μM, [ODN] = 1 mM strand in 50 mM phosphate buffered D2O (pH 7). Recorded in 50 μM pathlength liquid demountable cell (Harrick Corp.).

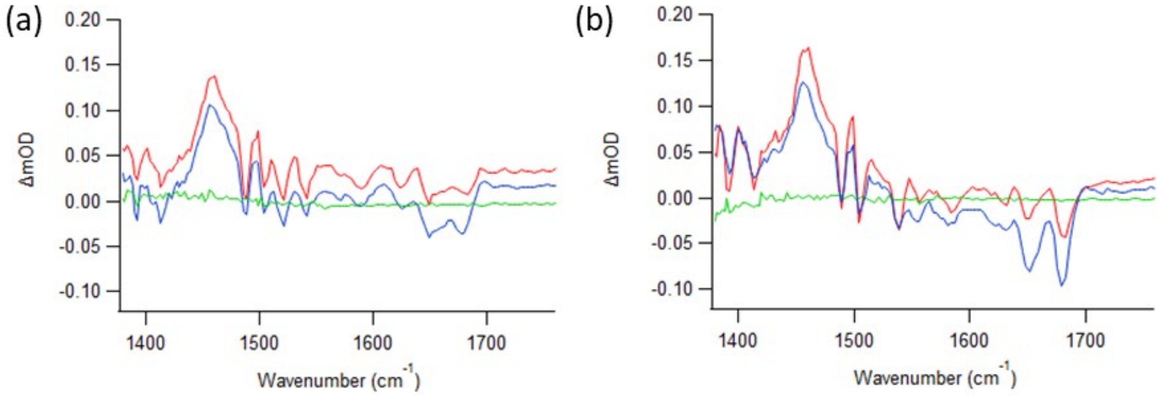

Fig. S7. Ps-TRIR spectra {at -100 ps (green); 20 ps (red) and 2000 ps (blue)} of (a) Λ-**1** (b) Δ-**1** in the presence of d({GC}$_5$)$_2$ in D_2O (50 mM phosphate pH 7). λ_{exc} = 400 nm.

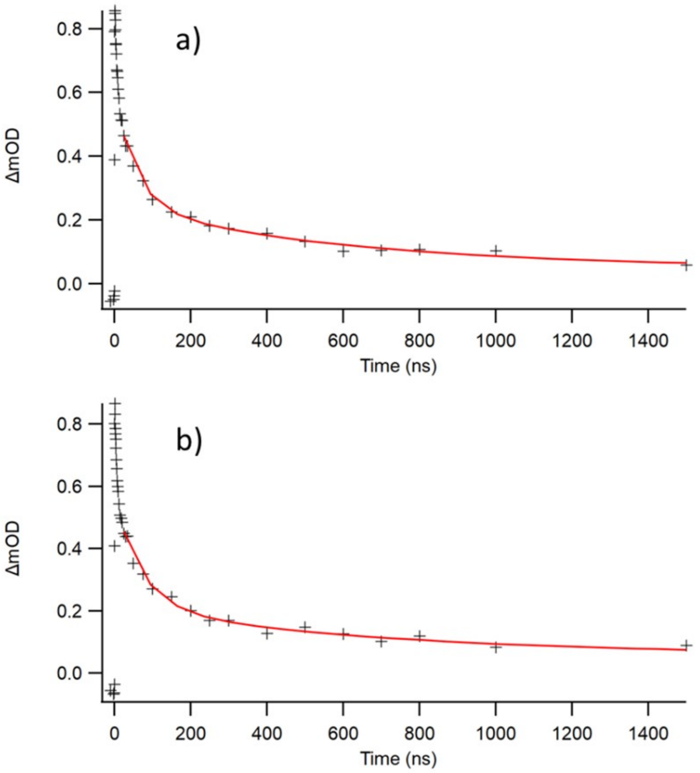

Fig. S8. Biexponential fits at 650 nm for (a) Λ-**1** + st-DNA (b) Δ-**1** + st-DNA in D_2O (50 mM phosphate pH 7). Delays fitted after 25 ps.

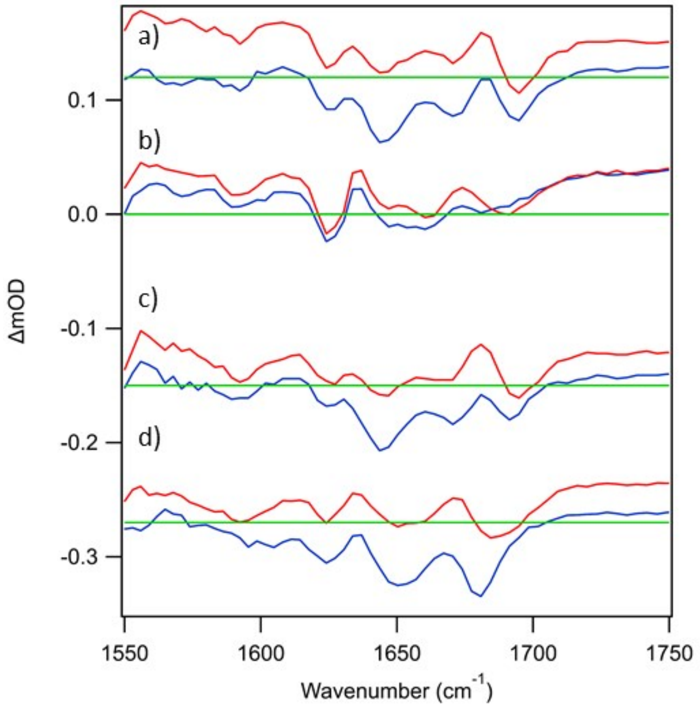

Fig S9. Ps-TRIR spectra (a) Λ-**1** + st-DNA (b) linear combination: 58% {Λ-**1** + poly(dA-dT)} + 42% {Λ-**1** + poly(dG-dC)} (c) Δ-**1** + st-DNA (d) 58% {Δ-**1** + poly(dA-dT)} + 42% {Δ-**1** + poly(dG-dC)}. Spectra shown at 20 ps (red), 2000 ps (blue) and ΔOD = 0 (green). [Ru] = 400 µM. Linear combinations performed on spectra from Figs 3 & 4 in main paper.

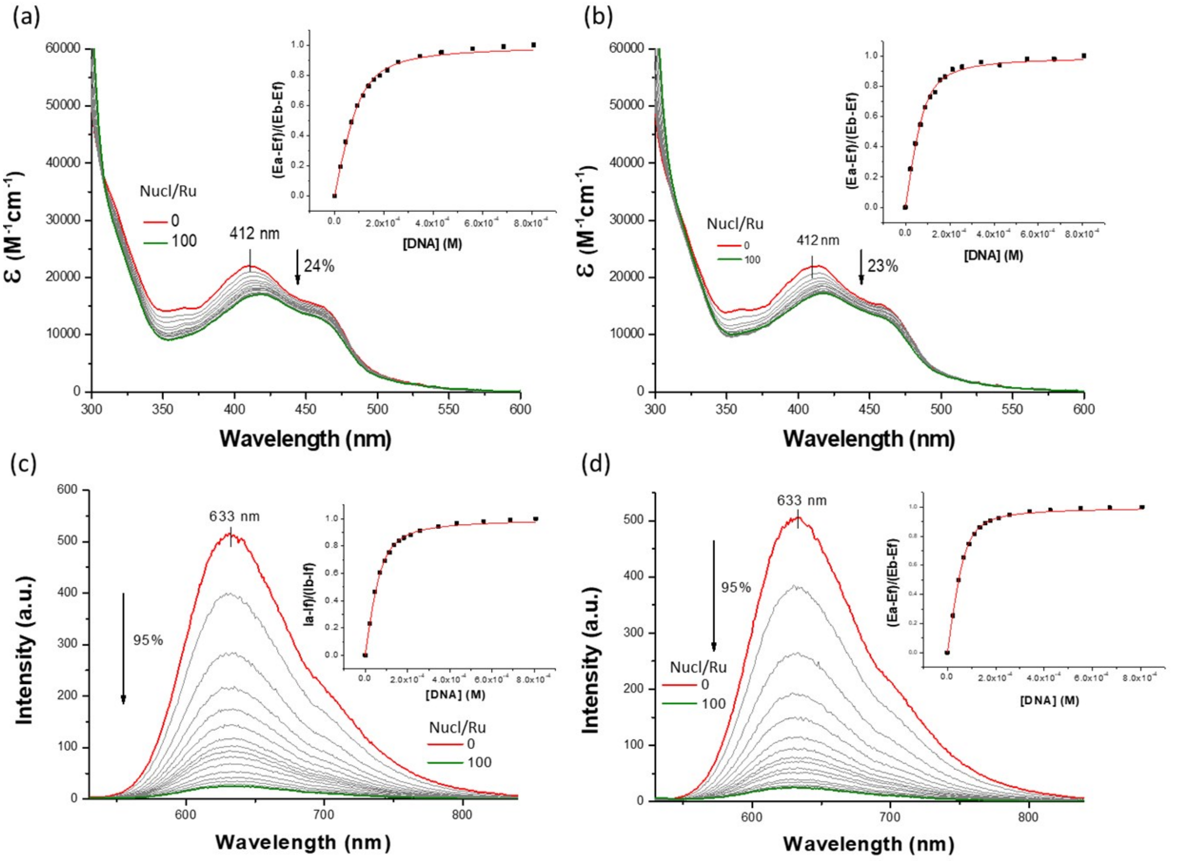

Fig. S10. Changes in the molar absorption coefficient of Δ-**1** (8.7 μM) with increasing additions of (a) d(CCGGATCCGG)$_2$ (0 – 807 μM) and (b) d(CCGGTACCGG)$_2$ (0 – 807 μM). Insert: Plots of (e_a-e_f)/(e_b-e_f) at 412 nm vs. [DNA] using data with [Nuc]/[Ru] between 0-100 and the best fits of the data (---) using the method of Carter et al.[1] Changes in the emission spectrum of Δ-**1** (8.7 μM) (λ_{ex} = 435 nm) with increasing additions of (c) d(CCGGATCCGG)$_2$ (0 – 807 μM) and (d) d(CCGGTACCGG)$_2$ (0 – 807 μM) in 50 mM K-phosphate buffer (pH 7.0) at 298 K. Inserts: Plots of (I_a-I_f)/(I_b-I_f) at 633 nm vs. [DNA] using data with [Nuc]/[Ru] between 0 – 100 and the best fits of the data (---) using the method of Carter et al.[1] DNA concentrations expressed in nucleotides. All data in aerated 50 mM K-phosphate buffer (pH. 7.0) at 298 K.

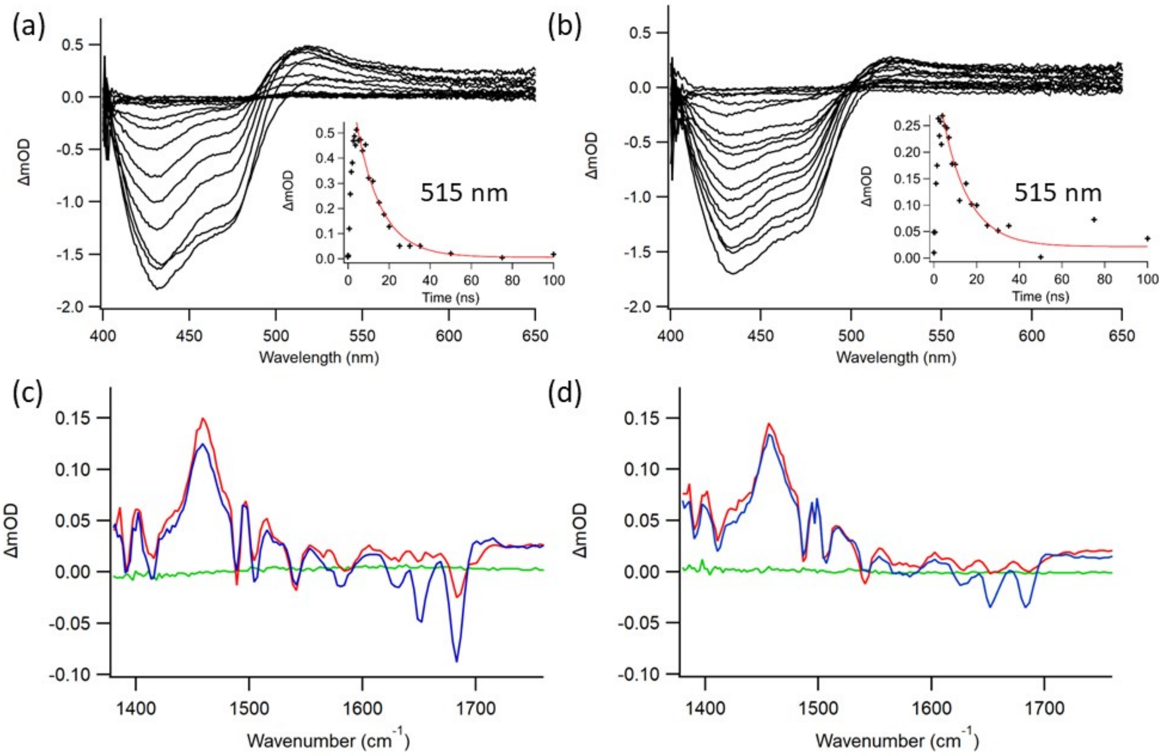

Fig. S11. Ns-TrA spectra of Δ-**1** in the presence of (a) d(CCGGATCCGG)$_2$ (b) d(CCGGTACCGG)$_2$. Inserts: monoexponential fits at 515 nm (τ = 12 ±1 ns and 12 ± 2 ns, resp.). λ_{exc} = 355 nm. Spectra shown from 0.5 ns to 1000 ns. Ps-TRIR spectra {at -100 ps (green); 20 ps (red) and 2000 ps (blue)} of Δ-**1** in the presence of (c) d(CCGGATCCGG)$_2$ (d) d(CCGGTACCGG)$_2$. λ_{exc} = 400 nm. All in D_2O 50 mM phosphate pH 7.

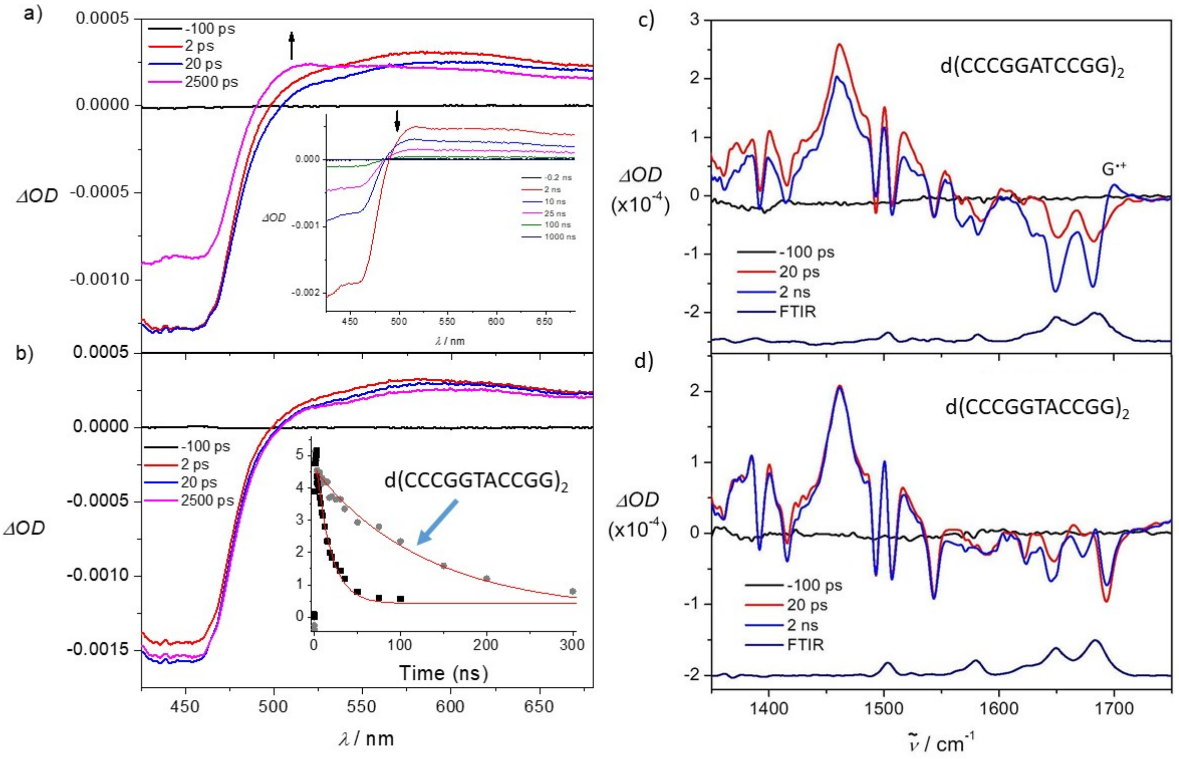

Fig S12. (a) Ps-TrA spectra of Λ-**1** in the presence of d(CCGGATCCGG)$_2$. Insert: ns-TrA spectra at selected delays (b) Ps-TrA spectra of Λ-**1** in the presence of d(CCGGTACCGG)$_2$. Insert: Comparative Ns-TrA decay kinetics for Λ-**1** bound to d(CCGGATCCGG)$_2$ (black squares) and d(CCGGTACCGG)$_2$ (grey circles). Ps-TRIR spectra of Λ-**1** in the presence of (c) d(CCGGATCCGG)$_2$, (d) d(CCGGTACCGG)$_2$. λ_{exc} = 400 nm (1 μJ) in 50 mM phosphate buffered-D_2O [modified from Keane *et al. Angew. Chem. Int Ed.*, 2015, **54**, 8364–8368].[2]

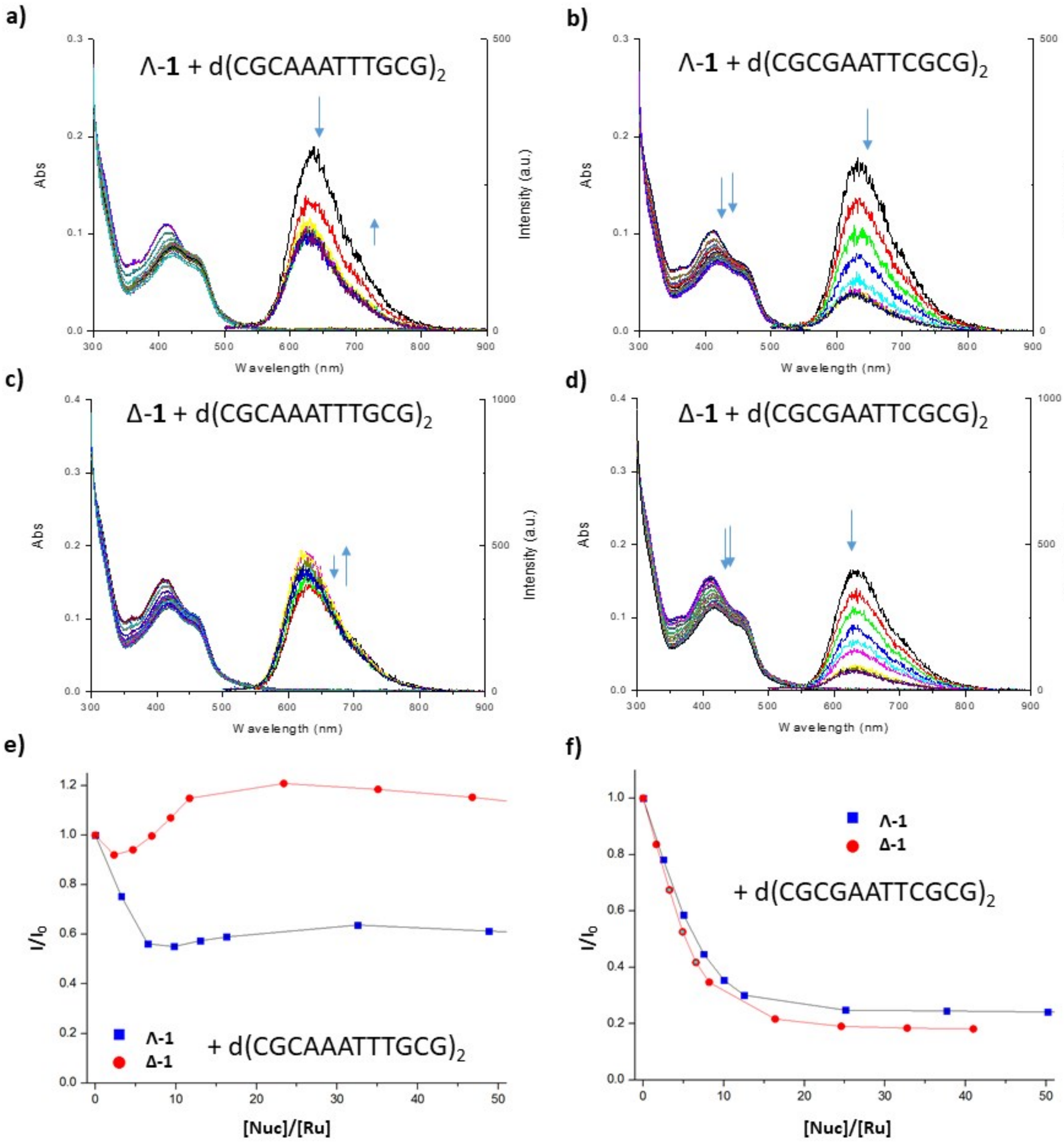

Fig. S13. Absorption and emission spectra of (a) Λ-**1** + d(CGCAAATTTGCG)$_2$ (b) Λ-**1** + d(CGCGAATTCGCG)$_2$ (c) Δ-**1** + d(CGCAAATTTGCG)$_2$ (d) Δ-**1** + d(CGCGAATTCGCG)$_2$. Changes in emission intensity (λ_{exc} = 415 nm) of Λ/Δ-**1** in the presence of (e) d(CGCAAATTTGCG)$_2$ (f) d(CGCGAATTCGCG)$_2$. In 50 mM K-phosphate buffer pH 7, [Ru] = 10 μM.

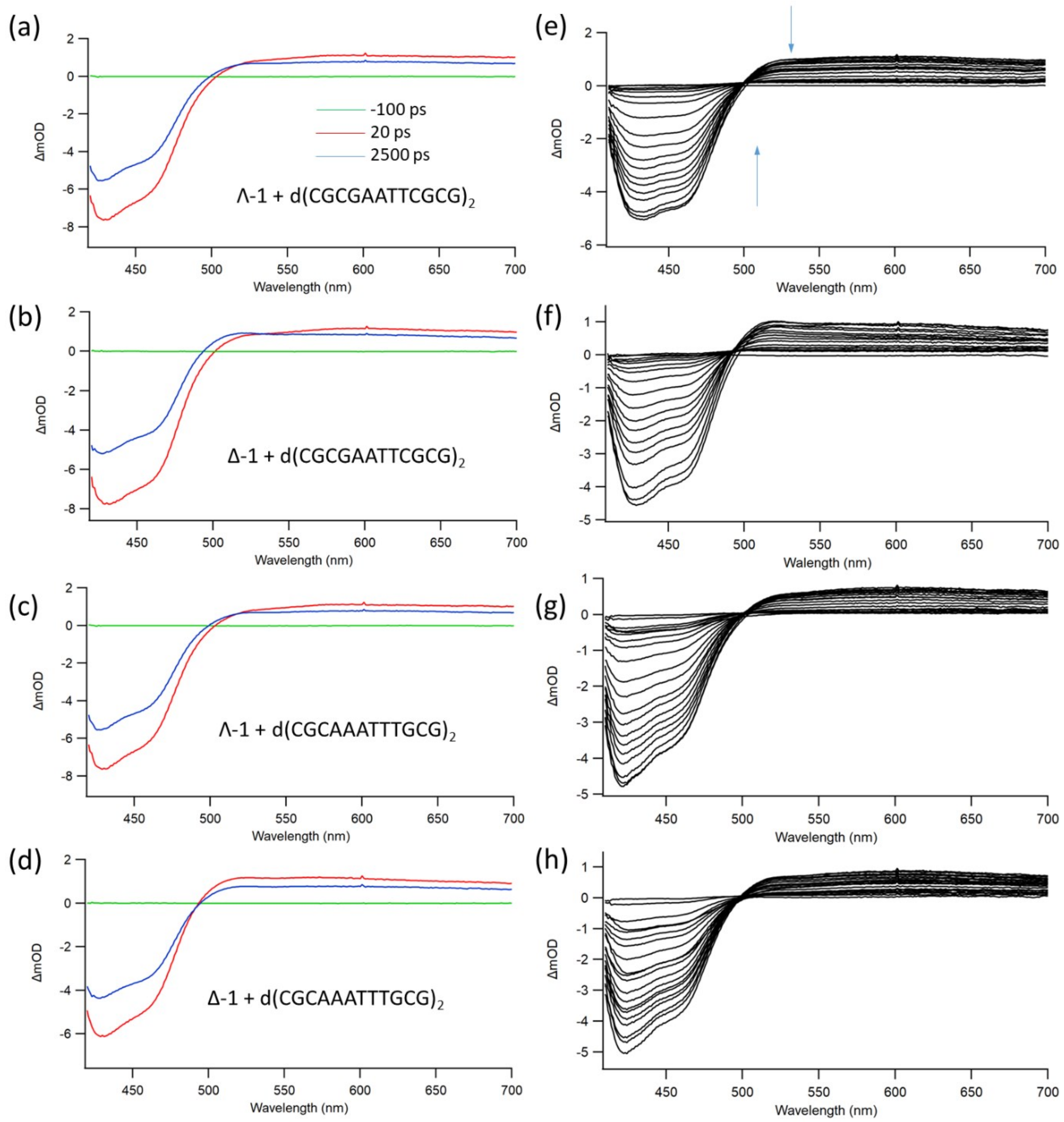

Fig. S14. Ps-TrA spectra of Λ/Δ-**1** in the presence of dodecamer ODNs at -100 ps (green) 20 ps (red) and 2500 ps (blue) following 400 nm excitation (a) Λ-**1** + d(CGCGAATTCGCG)$_2$ (b) Δ-**1** + d(CGCGAATTCGCG)$_2$ (c) Λ-**1** + d(CGCAAATTTGCG)$_2$ (d) Δ-**1** + d(CGCAAATTTGCG)$_2$; ns-TrA spectra of Λ/Δ-**1** in the presence of dodecamer ODNs at various delays (-10 ns to 5000 ns) following 355 nm excitation; (e) Λ-**1** + d(CGCGAATTCGCG)$_2$ (f) Δ-**1** + d(CGCGAATTCGCG)$_2$ (g) Λ-**1** + d(CGCAAATTTGCG)$_2$ (h) Δ-**1** + d(CGCAAATTTGCG)$_2$. [Ru] = 400 μM, [DNA] = 500 μM duplex in 50 mM phosphate buffer in D_2O.

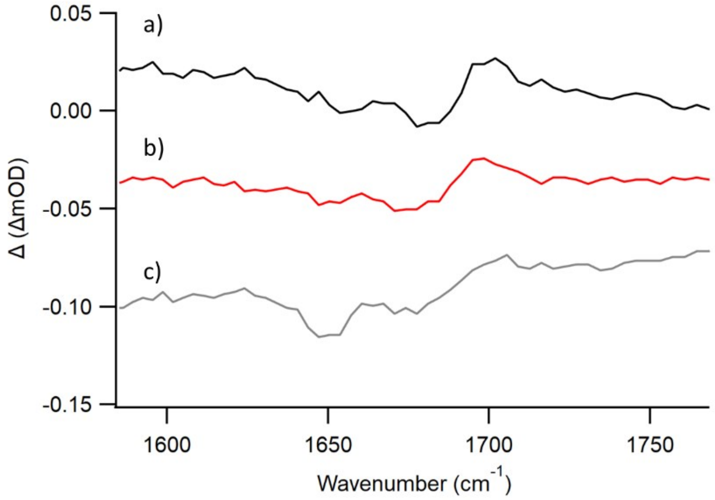

Fig. S15. TRIR subtraction spectra (2000 ps minus 20 ps) of Λ-**1** in the presence of mixed sequence DNAs (a) d(CGCGAATTCGCG)$_2$ (b) d(CGCAAATTTGCG)$_2$ (c) st-DNA.

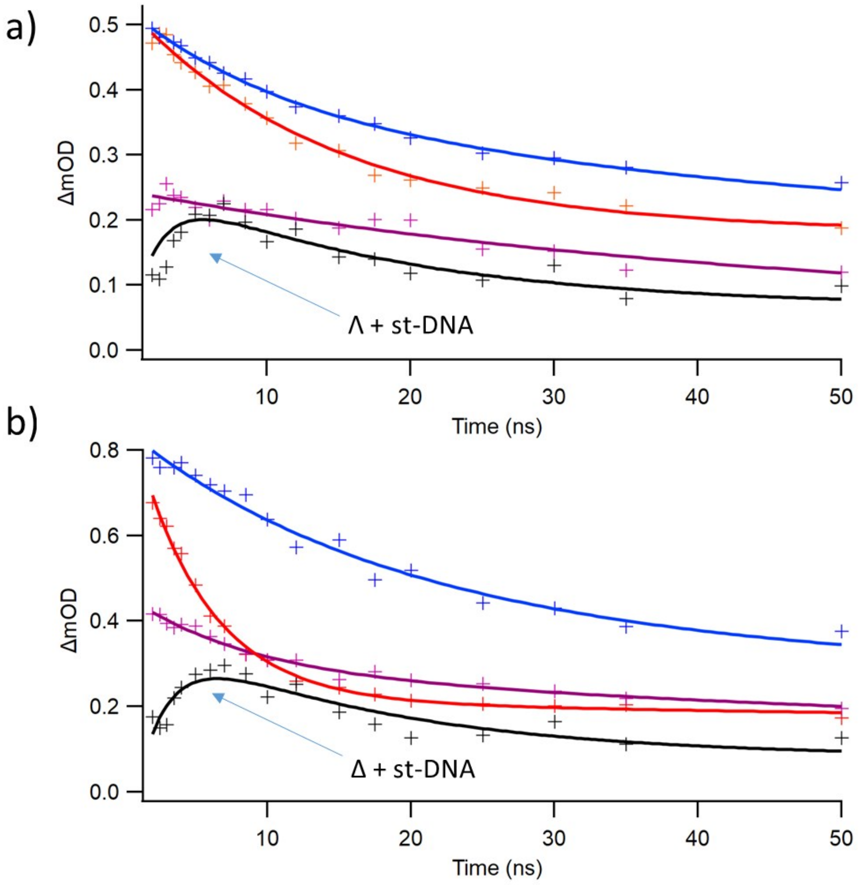

Fig. S16. Comparison of Ns-TrA kinetics at 507 nm of (a) Λ-**1** (b) Δ-**1** bound to selected poly- and oligo-deoxynucleotides. Red = poly(dG-dC); blue = d(CGCGAATTCGCG)$_2$; purple = d(CGCAAATTTGCG)$_2$; black = st-DNA. All in aerated 50 mM phosphate buffer in D_2O except poly(dG-dC) in 50 mM phosphate buffer in H_2O.

Tables

Table S1. Comparison of kinetics obtained from ps/ns-TrA data of Λ/Δ-**1** in the presence of poly(dG-dC) and d(GCGCGCGCGC)$_2$ in 50 mM phosphate buffer pH 7 in D_2O

**complex**	**ps-TrA**	**ns-TrA**
Λ-**1** + poly(dG-dC)	570 ± 60 ps	14.1 ± 2 ns
Δ-**1** + poly(dG-dC)	520 ± 50 ps	7.7 ± 0.8 ns
Λ-**1** + d({GC}$_5$)$_2$	590 ± 140 ps	12.2 ± 2.0 ns
Δ-**1** + d({GC}$_5$)$_2$	670 ± 70 ps	7.5 ± 0.8 ns

Table S2. Ns-TrA exponential fits of Λ/Δ-**1** in the presence of salmon-testes DNA

**complex**	**sequence**	**λ (nm)**	**τ_{growth} (ns)**	**τ_1 (ns)**	**A_1 (%)**	**τ_2 (ns)**	**A_2 (%)**
Λ-**1**	st-DNA	507	2.0 ± 0.5	14 ± 2	77	790 ± 330	23
Δ-**1**	st-DNA	507	2.0 ± 0.5	13 ± 2	72	610 ± 190	28
Λ-**1**	st-DNA	650[a]	-	57 ± 9	54	600 ± 90	46
Δ-**1**	st-DNA	650[a]	-	67 ± 10	62	680 ± 170	38

[a]Fit has been performed from 25 ns

Table S3. Average ns-TrA lifetimes (measured at 515 nm) of Λ/Δ-**1** in the presence of mixed sequence DNA(s)

**complex**	**sequence**	**τ_{ave}[a] (ns)**	**τ_1 (ns)**	**A_1 (%)**	**τ_2 (ns)**	**A_2 (%)**
Λ-**1**	d(CGCGAATTCGCG)$_2$	63.4	10.8	49	113	51
Δ-**1**	d(CGCGAATTCGCG)$_2$	69.7	14.8	62	161	38
Λ-**1**	d(CGCAAATTTGCG)$_2$	117.6	28.3	71	338	29
Δ-**1**	d(CGCAAATTTGCG)$_2$	125.4	16.7	52	243	48

[a]$\tau_{ave} = (A_1\tau_1+A_2\tau_2)/100$

Table S4. Average ns-TrA lifetimes (measured at 650 nm) of Λ/Δ-**1** in the presence of mixed sequence DNA(s).

**complex**	**sequence**	**τ_{ave}[a] (ns)**	**τ_1 (ns)**	**A_1 (%)**	**τ_2 (ns)**	**A_2 (%)**
Λ-**1**	d(CGCGAATTCGCG)$_2$	65.1	6.16	38	101	62
Δ-**1**	d(CGCGAATTCGCG)$_2$	55.4	5.47	46	98.1	54
Λ-**1**	d(CGCAAATTTGCG)$_2$	124.4	18.3	59	280	41
Δ-**1**	d(CGCAAATTTGCG)$_2$	223.2	14.8	51	438	49

[a]$\tau_{ave} = (A_1\tau_1+A_2\tau_2)/100$

Experimental

$[Ru(TAP)_2(dppz)]^{2+}$ (**1**) enantiomers were synthesised and resolved as below [reproduced from the Supporting Information of Keane *et al.*, *J. Phys. Chem. Lett.*, 2015, **6**, 734–738].[3]

The synthesis of *rac*-$[Ru(TAP)_2(dppz)]2Cl$ was carried out by a modification to the method previously described by Elias *et al.*[4] $Ru(TAP)_2Cl_2$ (0.155 g, 0.27 mmol, 1 eq) and dipyrido[3,2-*a*:2′,3′-*c*]phenazine (dppz) (0.076 g, 0.27 mmol, 1 eq) were suspended in $EtOH:H_2O$ (1:1 ratio, 16 mL) and this solution was degassed by bubbling with argon for 20 mins. The mixture was then stirred and heated at 140 ˚C for 40 mins using microwave irradiation. The resulting solution was filtered and the PF_6 salt of the complex was precipitated from the filtrate by addition of a concentrated aqueous solution of NH_4PF6. The resulting precipitate was isolated by centrifugation and washed with H_2O (5 mL x 2). The solid was dissolved in acetonitrile and purified by silica gel flash chromatography, eluting with CH_3CN/H_2O/aq. $NaNO_3$(sat.) (40:4:1) (R_f = 0.16). The PF_6 salt of the complex was again precipitated and washed as described above. The Cl complex was reformed by swirling of the complex in MeOH (20 mL) in the presence of Amberlite ion exchange resin (chloride form) for 40 mins. This suspension was filtered and the solvent removed under reduced pressure. The complex was then dissolved in H_2O and purified on an aqueous SP-Sephadex C-25 column, eluting with a gradient of aqueous NaCl (0.1-0.2 M). The PF_6 salt of the complex was again precipitated and washed as before. The chloride form was regenerated from Amberlite and dried under high vacuum to yield a red-brown solid (0.097 g, 44%).

1H NMR (600 MHz, D_2O, δ) 9.74 (dd, *J* = 8.3, 1.2 Hz, 2H) , 9.01 (d, *J* = 2.9 Hz, 2H), 8.99 (d, *J* = 2.8 Hz, 2H), 8.65 (s, 4H), 8.50 (d, *J* = 2.9 Hz, 2H), 8.46 (dd, *J* = 6.6, 3.4 Hz, 2H), 8.39 (d, *J* = 2.8 Hz, 2H), 8.20 (dd, *J* = 5.4, 1.2 Hz, 2H), 8.11 (dd, *J* = 6.6, 3.4 Hz, 2H), 7.88 (dd, *J* = 8.3, 5.4 Hz, 2H).13C NMR (151 MHz, D2O, δ) 153.9, 149.8, 149.0, 148.8, 148.5, 148.4, 145.0, 144.9, 142.52, 142.50, 142.4, 139.5, 135.4, 132.8, 132.60, 132.55, 130.9, 128.9, 127.4. HRMS-MALDI+ (*m/z*): [M]+ calcd. for C38H22N12Ru, 748.1134; found, 748.1154.

Resolution of the enantiomers of $[Ru(TAP)_2(dppz)]2Cl$ was achieved by a modification to the literature method reported by Vasudevan *et al.*[5] The complex was recycled through a 1 metre cation exchange column of CM-Sephadex C-25, eluted with aqueous (−)-O,O′-dibenzoyl-L-tartrate (0.1 M) until the Δ and Λ enantiomers were observed as two separate bands on the column, with the Λ enantiomer eluting first. The tartrate salt of the complexes obtained was converted to the chloride by passing the aqueous solution of each enantiomer through a column of Amberlite ion exchange resin (chloride form). Identification of each enantiomer was achieved by CD spectroscopy.[6] All chemicals were obtained from Sigma-Aldrich, Alfa Aesar or TCI and unless specified, were used without further purification. Amberlite IRA-400 (chloride form) resin was soaked in methanol (HPLC) and washed thoroughly with methanol and water before use. Deuterated solvents for NMR use were purchased from Apollo Ltd. Analytical TLC was performed using Merck Kieselgel 60 F254 silica gel plates. Chromatographic columns were run using Silica gel 60 (230-400 mesh ASTM). The NMR was recorded using an AV-600 spectrometer, operating at 600.1 MHz for 1H NMR and 150.6 MHz for 13C NMR. Shifts are referenced relative to the internal solvent signals. Mass spectra were recorded on a MALDI QToF Premier, running Mass Lynx NT V 3.4 on a Waters 600 controller connected to a 996 photodiode array detector with HPLC-grade methanol or acetonitrile. High resolution mass spectra were determined by a peak matching method, using glu-fib, as the standard reference (*m/z* = 1570.677). All accurate masses were reported within ± 5 ppm.

Samples of [poly(dA-dT)] and [poly(dG-dC)] were purchased from Amersham Biosciences or Sigma-Aldrich. Samples of salmon testes DNA (Sigma-Aldrich) were prepared by dissolving in sodium phosphate buffer (10 mM, pH 7.4, 1 mL) overnight and then either vortexed and centrifuged at 10,000 rpm for 20 minutes to remove any insoluble impurities or passed through a syringe filter (0.45 µm pore size, hydrophilic polyethersulfone membrane, PALL Acrodisc®). Such solutions of st-DNA gave a ratio of UV absorbance at 260 nm and 280 nm of 1.87:1. For transient spectroscopy experiments samples were additionally sonicated with a sonic tip/probe on ice. Oligodeoxynucleotides $d(GCGCGCGCGC)_2$, $d(CCGGATCCGG)_2$, $d(CCGGTACCGG)_2$, $d(CGCGAATTCGCG)_2$ and $d(CGCAAATTTGCG)_2$ were purchased from ATDBio and purified by gel filtration.

UV/vis absorption spectra were recorded on either a Varian Cary 200 or a Shimadzu UV-2401 PC spectrophotometer. Steady-state luminescence spectra were recorded on a Varian Cary Eclipse spectrofluorimeter. Ps/ns TrA/TRIR spectra were recorded on the ULTRA apparatus at the Lasers for Science Facility at the Rutherford Appleton Laboratories (Oxfordshire, UK) using procedures identical to those described in detail elsewhere.[3,7] Ps-TrA/TRIR experiments were recorded using 400 nm excitation at 1 µJ pulse energy, a pulse width of 150 fs and pump spot size of 100-150 µm. Ns-TrA experiments were performed using 355 nm excitation with a pulse width of approx. 1 ns and an energy of 1 µJ. All experiments were carried out in aerated solution in 50 mM potassium phosphate buffered D_2O solution (prepared by dissolving equimolar amounts of K_2HPO_4 and KH_2PO_4 in D_2O).

Single and double exponential kinetics were calculated from the change in transient absorption at single wavelengths using the Levenburg-Marquardt algorithm. Binding constants (K_b) and binding site size (n) for Λ/Δ-$[Ru(TAP)_2(dppz)]^{2+}$ with poly(dG-dC) in 10 mM phosphate buffer were calculated from emission titration data using the method of McGhee and von Hippel.[8] Fitting in the presence of $d(CCGGATCCGG)_2$ and $d(CCGGTACCGG)_2$ was made using the methods of Carter, Rodriguez and Bard[1] and those with $d(CGCGAATTCGCG)_2$ and $d(CGCAAATTTGCG)_2$ were calculated using the method of Poulsen et al.[9]

References

1. M. T. Carter, M. Rodriguez and A. J. Bard, *J. Am. Chem. Soc.*, 1989, **111**, 8901–8911.
2. P. M. Keane, F. E. Poynton, J. P. Hall, I. V. Sazanovich, M. Towrie, T. Gunnlaugsson, S. J. Quinn, C. J. Cardin and J. M. Kelly, *Angew. Chem., Int. Ed.*, 2015, **54**, 8364–8368.
3. P. M. Keane, F. E. Poynton, J. P. Hall, I. P. Clark, I. V. Sazanovich, M. Towrie, T. Gunnlaugsson, S. J. Quinn, C. J. Cardin and J. M. Kelly, *J. Phys. Chem. Lett.*, 2015, **6**, 734–738.
4. I. Ortmans, B. Elias, J. M. Kelly, C. Moucheron and A. Kirsch-DeMesmaeker, *Dalton Trans.*, 2004, 668-676.
5. S. Vasudevan, J. A. Smith, M. Wojdyla, A. di Trapani, P. E. Kruger, T. McCabe, N. C. Fletcher, S. J. Quinn and J. M. Kelly, *Dalton Trans.*, 2010, **39**, 3990-3998.
6. K. O'Sullivan, Ph.D. Thesis, University of Dublin, 2011.
7. S. J. Devereux, P. M. Keane, S. Vasudevan, I. V. Sazanovich, M. Towrie, Q. Cao, X.-Z. Sun, M. W. George, C. J. Cardin, N. A. P. Kane-Maguire, J. M. Kelly and S. J. Quinn, *Dalton Trans.*, 2014, **43**, 17606–17609.
8. J. D. McGhee and P. H. von Hippel, *J. Mol. Biol.*, 1974, **86**, 469-489.
9. B. C. Poulsen, S. Estalayo-Adrián, S. Blasco, S. A. Bright, J. M. Kelly, D. C. Williams and T. Gunnlaugsson, *Dalton Trans.*, 2016, **45**, 18208–18220.
